# Syntheses and Properties of Pentafluoroethylplumbanes in the Oxidation States +II, +III, and +IV

**DOI:** 10.1002/chem.71402

**Published:** 2026-07-18

**Authors:** Michael Grasse, Lukas Hartmann, Beate Neumann, Hans‐Georg Stammler, Berthold Hoge

**Affiliations:** ^1^ Fakultät für Chemie Centrum für Molekulare Materialien Universität Bielefeld Bielefeld Germany

**Keywords:** fluorine, lead, lead NMR, pentafluoroethyl lead compounds

## Abstract

Mono‐, bis‐, and tris(pentafluoroethyl)plumbanes of the type Pb(C_2_F_5_)*_n_* Ar_4‐_ *_n_* (Ar = Ph, Tol, Ani; *n* = 1–3) are easily accessible by treatment of the corresponding arylchloroplumbanes with LiC_2_F_5_. The subsequent treatment with HCl furnishes chloro(pentafluoroethyl)plumbanes. The synthesis of the tris(pentafluoroethyl)plumbate(II) anion is facilitated by treatment of Pb(C_2_F_5_)_3_Cl with PPh_3_. In addition, formation of Pb(C_2_F_5_)_4_ is achieved starting from Pb(OAc)_4_ and is fully characterized by multinuclear NMR spectroscopy and by single crystal x‐ray diffraction of the 1,10‐phenanthroline adduct.

## Graphical abstract

Syntheses of pentafluoroethyl substituted plumbanes in the formal oxidation states +II, +III, and +IV are reported. The compounds were characterized by multinuclear NMR spectroscopy, including ^207^Pb NMR spectroscopy, mass spectrometry and single‐crystal x‐ray diffraction experiments.

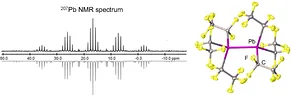

## 1 Introduction

Even though lead had and still has some very well‐known uses in the history of mankind, the toxicity of lead deters many scientists from researching lead compounds. The Romans used lead for water pipes, as lead is ductive and noncorrosive. Since the 1920s, lead has been widely used as an octane rating booster in gasoline, primarily in the form of tetraethyllead [1, 2], until it was replaced by dialkyl ether derivatives, such as methyl tertiary‐butyl ether (MTBE), or aromatic compounds, such as benzene, in the 1980s. Today lead is still used in lead–acid batteries. Despite the toxicity, there is still progress in the lead chemistry, mostly concerning the use in high‐performance perovskite‐sensitized solar cells [3].

The synthesis and chemistry of perfluoroalkyl lead compounds are not well studied. The first systematic study of trifluoromethylplumbanes was performed by Patorra and Eujen in the early 1990s [4, 5]. Pb(CF_3_)_4_ was first prepared by Juhlke and Lagow [6] on a milligram scale by reacting PbCl_2_ with CF_3_ radicals. The first pentafluoroethylplumbane, Pb(C_2_F_5_)Me_3_, was reported in 1960 [7, 8], but no spectroscopic evidence was given. Lange and Naumann [9] prepared Pb(C_2_F_5_)Me_3_ by reaction of Cd(C_2_F_5_)_2_·donor with Pb(OCOCF_3_)Me_3_ and studied it via NMR spectroscopy.

The C_2_F_5_ group shows a significantly higher thermal stability than the CF_3_ group. This can be impressively demonstrated for the corresponding silanes. Si(CF_3_)_3_NEt_2_ is unstable at room temperature and decomposes violently [10, 11]. In contrast to that Si(C_2_F_5_)_3_NEt_2_ [12] is stable up to 180°C for more than 2 h. C_2_F_5_ groups can be easily introduced with LiC_2_F_5_ [13, 14, 15]. This organometallic compound is, in contrast to LiCF_3_, stable in ethereal solution up to −40°C. It is easily accessible by the deprotonation of HC_2_F_5_ with *n*‐buthyllithium at temperatures around −78°C (Scheme 1).

**SCHEME 1:**
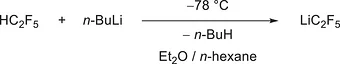
Synthesis of pentafluoroethyllithium by deprotonation of pentafluoroethane at low temperatures.

Employing freshly generated LiC_2_F_5_, a series of group IV and V element compounds have already been prepared in our group, for example Si(C_2_F_5_)_4_ [12], (Ge{C_2_F_5_}_3_)_2_O [16], Sn(C_2_F_5_)_3_H [17], and Bi(C_2_F_5_)_3_ [18].

In this work we present a series of mono‐, bis‐, tris‐, and tetrakis(pentafluoroethyl)plumbanes. They are prepared with high efficiency on a large scale along with detailed NMR spectroscopic examinations and solid state structure analyses [19].

Electron‐withdrawing perfluoroorganyl groups, such as the CF_3_ and C_2_F_5_ groups, behave as so‐called pseudohalogens, occupying a position between halogens and organyl groups in terms of their chemical properties. While lead(II) halides are significantly more stable than the corresponding lead(IV) halides, the opposite is true for organyl derivatives. In the context of our investigation into C_2_F_5_ lead derivatives, we aim to demonstrate that Pb(IV), Pb(III), and Pb(II) derivatives can be stabilized by electron‐withdrawing pentafluoroethyl groups, resulting in unusual redox behavior.

## 2 Results and Discussion

Tetrakis(pentafluoroethyl)plumbane could not be prepared via the reaction of LiC_2_F_5_ with lead(IV)‐chloride. PbCl_4_ decomposes slowly at room temperature and therefore must be freshly prepared by treatment of a [PbCl_6_]^2–^ salt with concentrated sulfuric acid [20]. The PbCl_4_ originating from this reaction still contains some acid causing the decomposition of LiC_2_F_5_. The use of Pb(OAc)_4_ circumvents this obstacle and tetrakis(pentafluoroethyl)plumbane can be obtained (Scheme 2).

**SCHEME 2:**
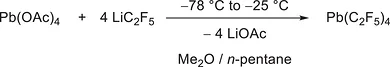
Synthesis of tetrakis(pentafluoroethyl)plumbane from lead(IV)acetate.

The classic reaction with LiC_2_F_5_ takes place in Et_2_O, which, however, could not be separated from the product. This is circumvented by using Me_2_O. Its low boiling point of −25°C enables the separation of the solvent and product. The yield of the reaction (28 %) is not satisfying, but still better than that for Pb(C_6_F_5_)_4_ from Pb(OAc)_4_ with LiC_6_F_5_ (15 %) [21]. The ^19^F NMR spectrum displays singlet resonances for both, the CF_2_ and the CF_3_ group. The CF_2_ group also reveals lead satellites with a ^2^ *J*(Pb,F) coupling constant of 651 Hz. The ^207^Pb NMR spectrum exhibits, due to coupling to the 8 (CF_2_), respectively 12 (CF_3_) equivalent fluorine atoms, a nonet of tridecets at −415.5 ppm (Figure 1).

**FIGURE 1:**
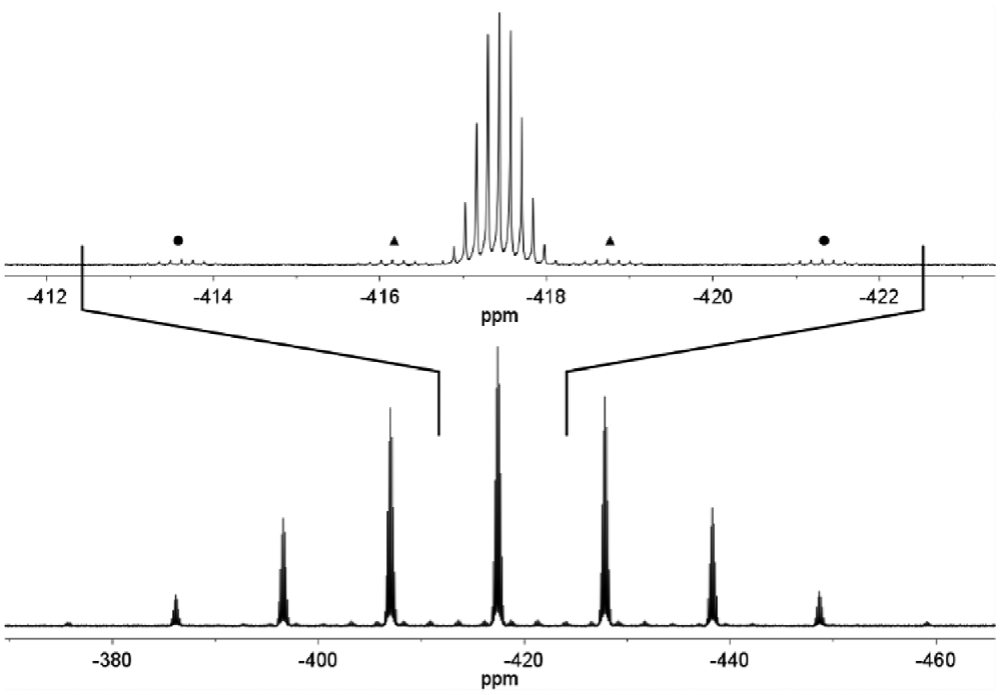
^207^Pb NMR spectrum of neat Pb(C_2_F_5_)_4_. Bottom: full spectrum; top: expansion of the central signal. ^13^C satellites on the central signal (^1^ *J*(Pb,C) = 480 Hz (●), ^2^ *J*(Pb,C) = 162 Hz (▲)).

The Pb(CF_3_)_4_ appears at −562.2 ppm in the ^207^Pb NMR spectrum [4]. In order to obtain the molecular structure, this novel lead compound is converted into the 1,10‐phenanthroline (phen) adduct. Recrystallisation from chloroform provided single crystals, suitable for an x‐ray structure analysis. [Pb(C_2_F_5_)_4_(phen)] crystallizes in the monoclinic space group *P2*_1_/*c*. The molecular structure in the solid state is shown in Figure 2 and provides the first example of a structurally characterized hexacoordinated tetraorganylplumbane containing a chelate ligand containing two nitrogen atoms.

**FIGURE 2:**
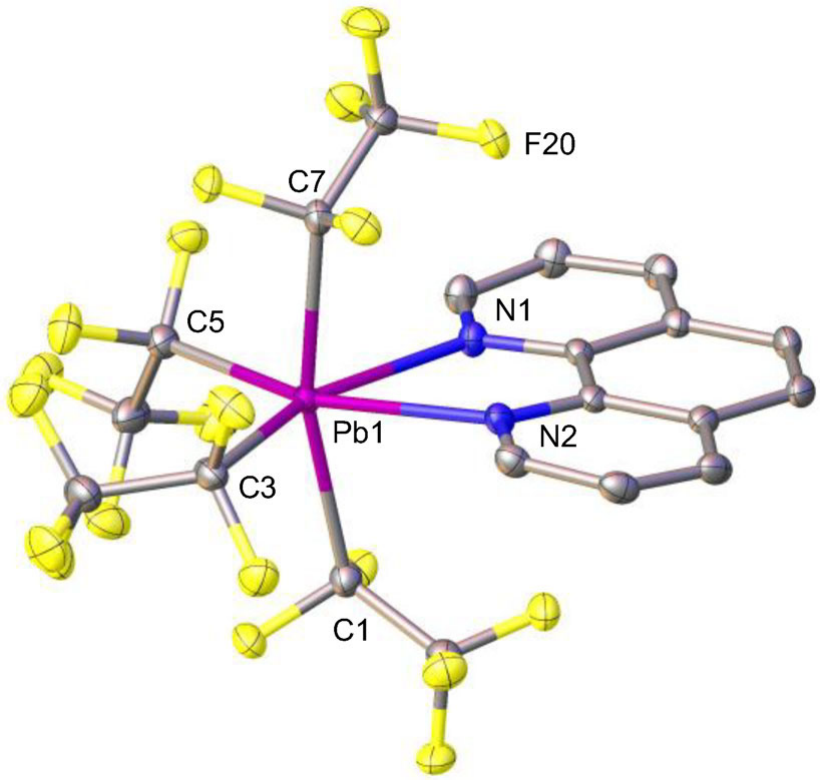
Molecular structure of [Pb(C_2_F_5_)_4_(phen)] (thermal ellipsoids are shown at the 50% probability level, hydrogen atoms omitted for clarity). Selected bond lengths [pm] and angles [°]: Pb1‐N1 254.7(2), Pb1‐N2 254.61(19), Pb1‐C1 234.5(2), Pb1‐C3 237.3(2), Pb1‐C5 238.1(2), Pb1‐C7 235.4(2), C1‐Pb1‐C7 165.04(8), C3‐Pb1‐C5 106.46(9), and N1‐Pb1‐N2 65.77(6).

The central lead atom exhibits a strongly distorted octahedral coordination sphere with a C1‐Pb1‐C7 bond angle of only 165°. The formally axial C_2_F_5_ groups, which are arranged in a trans configuration relative to one another, have a Pb─C bond length that is approximately three pm shorter than that of the cis‐arranged, formally equatorial C_2_F_5_ groups. The steric demand of the CF_2_ subunits opposite of the phenanthroline ligand pushes the two other C_2_F_5_ units into the direction of the ligand. The molecular structure indicates that the C_2_F_5_ groups are chemically inequivalent and thus two sets of signals are expected in the ^19^F NMR spectrum. At room temperature only one very broad resonance is observed. Low temperature NMR experiments at −50°C reveal two sets of signals for the C_2_F_5_ groups. The resonances for the CF_3_ units are observed at −80.8 and −81.9 ppm, respectively, while signals for the CF_2_ units are displayed at −101.0 (^2^ *J*(Pb,F) = 995 Hz) and −101.4 ppm (^2^ *J*(Pb,F) = 404 Hz). Compared to free Pb(C_2_F_5_)_4_, the absolute value of the ^2^ *J*(Pb,F) coupling constants changes by +344 and −247 Hz, respectively. In the ^207^Pb NMR spectrum, a significant high field shift from −416 ppm for Pb(C_2_F_5_)_4_ to −860 ppm for the phen adduct is observed and the resonance splits into a quintet of quintets.

In order to synthesize functionalized tris(pentafluoroethyl)plumbanes, it would be convenient to use halogens like Cl_2_ to cleave one lead carbon bond of Pb(C_2_F_5_)_4_ and generate Pb(C_2_F_5_)_3_Cl. However, only decomposition was observed under the applied reaction conditions. As a consequence, aryl substituents as protecting groups were employed. The syntheses of PbAr_4_ (Ar = Ph, Tol and Ani) via LiAr and ArI is well known in the literature (Scheme 3) [22, 23].

**SCHEME 3:**
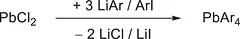
Syntheses of tetraarylplumbanes (Ar = C_6_H_5_, C_6_H_4_CH_3_, and C_6_H_4_OCH_3_).

The molecular structure of PbAni_4_ in the solid state has in contrast to PbPh_4_ [24, 25, 26] and PbTol_4_ [27] not been described before. PbAni_4_ crystallizes in the tetragonal space group *I*‐4. Figure 3 shows the *S*_4_ symmetrical molecule marked with the *S*_4_ axis.

**FIGURE 3:**
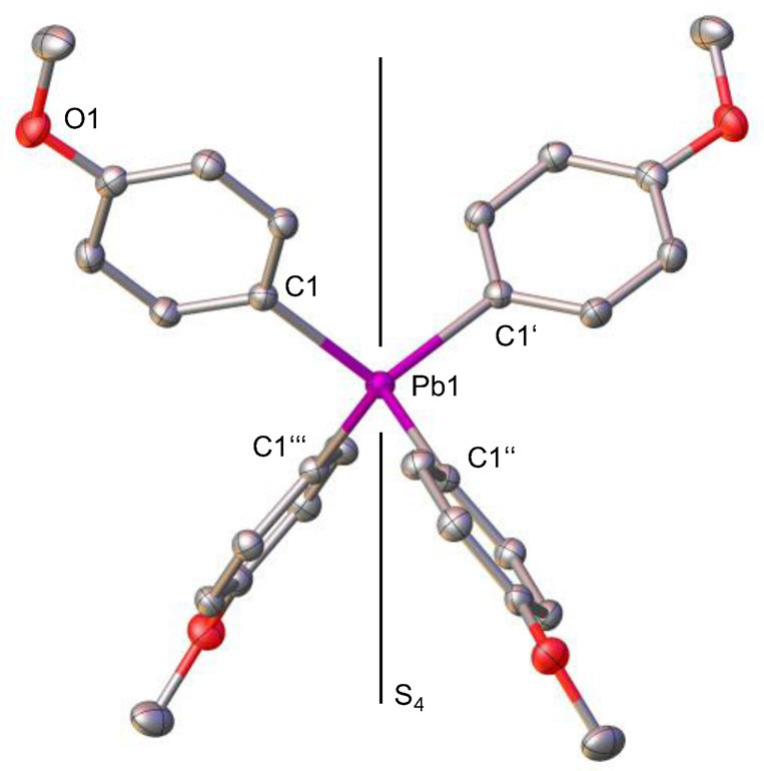
Molecular structure of PbAni_4_ with the *S*_4_ axis (ellipsoids set at 50 % probability, hydrogen atoms omitted for clarity). Selected bond lengths [pm] and angles [°]: 4x Pb1‐C1 220.5(2), C1─Pb1─C1′═C1″─Pb1─C1‴ 112.88(10), and C1─Pb1─C1″═C1─Pb1─C1‴═C1′─Pb1─C1″═C1′─Pb1─C1‴ 107.79(5).

The structure is best described as a tetrahedron compressed along the *S*_4_ axis, with C‐Pb‐C bond angles of 112.9° and 107.8°. These values and the Pb─C bond length are in good agreement with the literature values for other tetraarylplumbanes [24, 25, 26, 27, 28]. Reaction of PbAr_4_ with one or two equivalents of gaseous hydrogen chloride at ambient temperatures gave the desired compounds PbClAr_3_ and PbCl_2_Ar_2_ as off‐white solids, after removal of all volatile compounds *in vacuo* (Scheme 4).

**SCHEME 4:**
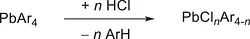
Syntheses of arylchloroplumbanes (Ar = Ph, Tol, and Ani).

Even by using slightly less than one equivalent of hydrogen chloride the triarylchloroplumbanes cannot be obtained selectively. Instead a mixture of PbClAr_3_, PbCl_2_Ar_2_, and PbAr_4_ is observed. The diaryldichloroplumbanes can prepared with high selectivity by using two or more equivalents of gaseous hydrogen chloride.

For the addition of C_2_F_5_ groups, LiC_2_F_5_ is prepared in situ at −78°C by the combination of *n*‐butyllithium with pentafluoroethane in a diethyl ether solution. An arylchloroplumbane is then added at low temperatures. Under these conditions the desired aryl(pentafluoroethyl)plumbanes are afforded with excellent yields on a large scale (Pb(C_2_F_5_)_2_Tol_2_, 95%, 30 g) (Scheme 5).

**SCHEME 5:**
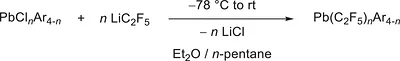
Syntheses of ary(pentafluoroethyl)plumbanes from diaryldichloroplumbanes (Ar = Ph, Tol, and Ani; *n* = 1, 2).

The diarylbis(pentafluoroethyl)plumbanes are viscous liquids with high boiling points. Pb(C_2_F_5_)_2_Ani_2_, for example, has a boiling point of 145°C at 1∙10^−6^ bar and can therefore be easily purified by high vacuum distillation. In contrast to that, Pb(C_2_F_5_)Ph_3_ and Pb(C_2_F_5_)Tol_3_ are colorless solids which are hardly soluble in cold *n*‐pentane. They can be purified from residual diarylbis(pentafluoroethyl)plumbanes by washing the crude product with the alkane.

Single crystals, suitable for an x‐ray structure analysis, of Pb(C_2_F_5_)Ph_3_ and Pb(C_2_F_5_)Tol_3_ can be obtained from *n*‐pentane solution by slow evaporation of the solvent. Pb(C_2_F_5_)Ph_3_ crystallizes in the triclinic space group *P*‐1, and Pb(C_2_F_5_)Tol_3_ crystallizes in the monoclinic space group *P*2_1_/*c* (Figure 4). Selected bond lengths and bond angles are given in Table 1. The asymmetric unit of the phenyl derivative comprises two molecules with a disorder in both pentafluoroethyl moieties. The tolyl derivate consists of only one molecule with no disorder.

**FIGURE 4:**
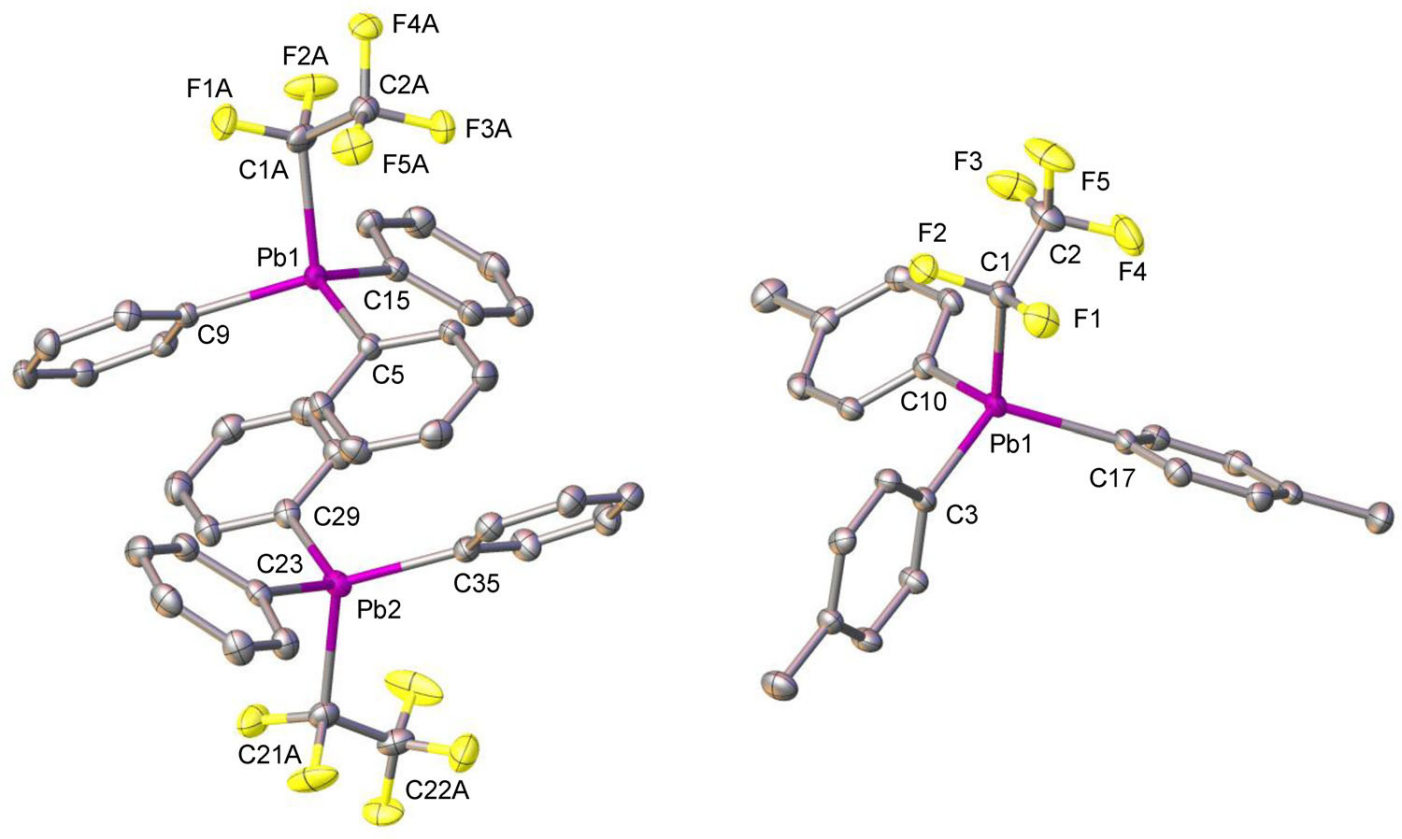
Molecular structures of Pb(C_2_F_5_)Ph_3_ and Pb(C_2_F_5_)Tol_3_ (thermal ellipsoids are shown at the 50% probability level, hydrogen atoms omitted for clarity). For Pb(C_2_F_5_)Ph_3_, the C_2_F_5_ groups at Pb1, respectively Pb2 are disordered over two sites by 28 and 40%, the minor occupied disordered parts are omitted.

**TABLE 1:** Selected bond lengths [pm] and bond angles [°] for Pb(C_2_F_5_)Ph_3_ (major structure) and Pb(C_2_F_5_)Tol_3_.

	Pb(C_2_F_5_)Ph_3_	Pb(C_2_F_5_)Tol_3_
Pb1─C1	231.5(9)	230.3(2)
Pb1─C3	219.2(2)	219.1(2)
Pb1─C9/C10	218.9(2)	219.9(2)
Pb1─C15/C17	218.5(2)	219.3(2)
C1─Pb1─C3	108.46(15)	102.30(7)
C1─Pb1─C9/C10	103.3(2)	109.83(7)
C1─Pb1─C15/C17	103.17(18)	106.89(7)

The molecular structures of Pb(C_2_F_5_)Ph_3_ and Pb(C_2_F_5_)Tol_3_ are very similar. In both cases, the central lead atoms reside in the anticipated tetrahedral geometry. The Pb1─C1 bond vectors are in both structures not orthogonal on a plane spanned through the three aromatic carbons bonded directly to the central lead atom. Instead, they are slanted by 3.5° (Pb(C_2_F_5_)Ph_3_) and 3.9° (Pb(C_2_F_5_)Tol_3_). This behavior takes the steric demand of the C_2_F_5_ moiety into account.

Treatment of diarylbis(pentafluoroethyl)plumbanes with HCl and subsequent pentafluoroethylation selectively affords the corresponding aryltris(pentafluoroethyl)plumbanes on a large scale (Pb(C_2_F_5_)_3_Tol, 94%, 27 g) (Scheme 6). The obtained lead compounds are colorless liquids with a significantly lower boiling point than their diarylbis(pentafluoroethyl)plumbane analogues (Pb(C_2_F_5_)_2_Tol_2_: 108°C, 1∙10^−6^ bar; Pb(C_2_F_5_)_3_Tol: 92°C, 7∙10^3^ bar). Even by using a large excess of HCl only one aryl group is cleaved. The use of HBr or the addition of AlCl_3_ or AlBr_3_, respectively, does not afford Pb(C_2_F_5_)_2_X_2_ (X = Cl, Br), in contrast to the corresponding germanium compound Ge(C_2_F_5_)_2_Br_2_ from Ge(C_2_F_5_)_2_Ph_2_ [29].

**SCHEME 6:**
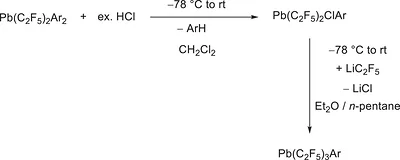
Syntheses of aryltris(pentafluoroethyl)plumbanes from diarylbis(pentafluoroethyl)plumbanes (Ar = Ph, Tol, and Ani).

Multinuclear NMR spectroscopy is an excellent tool for the characterization of aryl(pentafluoroethyl)plumbanes. Pb(C_2_F_5_)_2_Tol_2_, for example, can be fully characterized by NMR spectroscopy. All atoms in this compound have an isotope with a nuclear spin of *I* = 1/2 and are therefore easily observable by NMR. The NMR active isotope of lead is ^207^Pb with a natural abundance of 22.1%. Lead is the heaviest, nonradioactive element of the periodic table, which also makes ^207^Pb the heaviest stable isotope with a nuclear spin of *I* = 1/2 [20].

In Table 2, selected NMR spectroscopic parameters for a series of aryl(pentafluoroethyl)plumbanes are shown.

**TABLE 2:** Selected NMR spectroscopic parameters for a series of aryl(pentafluoroethyl)plumbanes, Pb(C_2_F_5_)*_n_* Ar_4‐_ *_n_* (*n* = 1–3, Ar = Ph, Tol, and Ani).

	Pb(C_2_F_5_)Ph_3_ ^a^	Pb(C_2_F_5_)Tol_3_ ^b^	Pb(C_2_F_5_)_2_Ph_2_ ^c^	Pb(C_2_F_5_)_2_Tol_2_ ^c^	Pb(C_2_F_5_)_2_Ani_2_ ^c^	Pb(C_2_F_5_)_3_Ph^c^	Pb(C_2_F_5_)_3_Tol^c^	Pb(C_2_F_5_)_3_Ani^d^
*δ*(^207^Pb)/ppm	−204	−181	−249	−233	−218	−319	−306	−292
*δ*(^19^F, CF_2_)/ppm	−110.6	−110.4	−106.8	−107.2	−107.7	−102.1	−102.3	−101.1
^2^ *J*(Pb,F)/Hz	324	329	408	398	400	515	511	508
^3^ *J*(Pb,F)/Hz	6	6	/	/	/	5	4	4
^3^ *J*(Pb,H)/Hz	93	93	110	110	105	131	132	125
^4^ *J*(Pb,H)/Hz	32	31	41	40	32	54	51	44
*^n^J*(Pb,H)/Hz	17	5	21	7	/	25	9	/

^a^ CD_2_Cl_2_.

^b^ CDCl_3_.

^c^ Neat/[D_6_]acetone.

^d^ C_6_D_6_.

The shift of the resonance of the lead atom in the ^207^Pb NMR spectrum is very sensitive toward the substitution with C_2_F_5_ groups.

With an increasing number of C_2_F_5_ groups a significant highfield shift is observed. For the tolyl substituted plumbanes, chemical shifts from *δ*(^207^Pb) = −181 ppm (Pb(C_2_F_5_)Tol_3_) to −360 ppm (Pb(C_2_F_5_)_3_Tol) are observed. The same trend can be observed for trifluoromethylplumbanes and pentafluorophenylplumbanes [30], which demonstrates an unusual shielding effect of perfluoroorganyl groups.

Due to the large ^2^ *J*(Pb,F) and *^n^J*(Pb,H) coupling constants the respective number of aryl and pentafluoroethyl substituents can be determined by the multiplicity in the ^207^Pb NMR spectrum. Figure 5 shows the ^207^Pb, ^207^Pb{^1^H}, and ^207^Pb{^19^F} NMR spectra of Pb(C_2_F_5_)_3_Tol.

**FIGURE 5:**
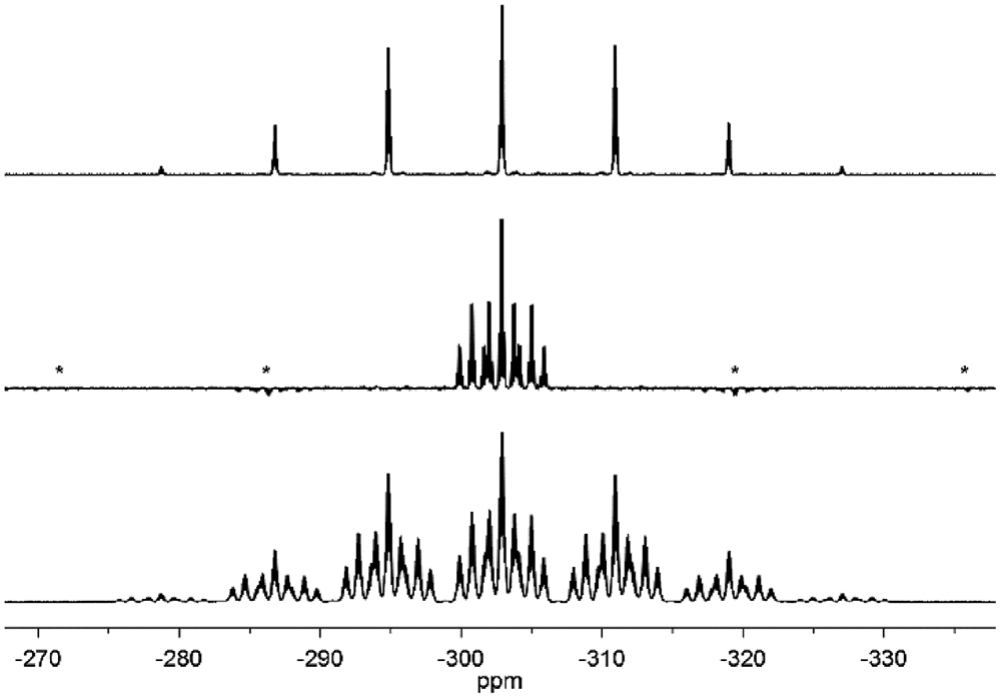
^207^Pb{^1^H} (top), ^207^Pb{^19^F} (middle), and ^207^Pb (bottom) NMR spectrum of Pb(C_2_F_5_)_3_Tol. Decoupling artefacts are marked (*).

The ^207^Pb{^19^F} NMR spectrum of Pb(C_2_F_5_)_3_Tol reveals a triplet of triplets of quartets at −306 ppm due to the three lead hydrogen couplings (^3^ *J*(Pb,H) = 132 Hz, ^4^ *J*(Pb,H) = 51 Hz, and ^6^ *J*(Pb,H) = 9 Hz). Due to large coupling constants and very large shift range, decoupling artefacts cannot be avoided. The ^207^Pb{^1^H} NMR shows a septet of decets due to the coupling to six fluorine atoms of three CF_2_ units (^2^ *J*(Pb,F) = 511 Hz) and to the nine fluorine atoms of three CF_3_ units (^3^ *J*(Pb,F) = 4 Hz). Consequently, the non‐decoupled ^207^Pb NMR spectrum reveals a septet of triplets of triplets of quartets of decets (see Figure 5 bottom). In the ^19^F NMR spectrum two singlets are observed at 84.5 ppm (CF_3_) and at −102.3 ppm with lead satellites (^2^ *J*(Pb,F) = 511 Hz, CF_2_). The ^1^H NMR spectrum is characterized by two doublets and a singlet, each of them with lead satellites. Remarkable are the lead satellites at the resonance for the CH_3_ group, due to a lead hydrogen coupling over six bonds (^6^ *J*(Pb,H) = 9 Hz).

To obtain the functionalized chlorotris(pentafluoroethyl)plumbane, the aryl derivatives were treated with gaseous HCl. While the reaction did not proceed selectively for Pb(C_2_F_5_)_3_Ph, Pb(C_2_F_5_)_3_Tol, and Pb(C_2_F_5_)_3_Ani were converted selectively into Pb(C_2_F_5_)_3_Cl, with the former being easier to handle in terms of synthesis and purification (Scheme 7).

**SCHEME 7:**
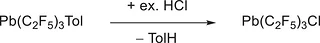
Synthesis of chlorotris(pentafluoroethyl)plumbane from tolyltris(pentafluoroethyl)plumbane.

Chlorotris(pentafluoroethyl)plumbane is synthesized via a solvent free reaction of tris(pentafluoroethyl)tolylplumbane with an excess of anhydrous hydrogen chloride. After 2 days of stirring at room temperature the chloroplumbane is obtained as large yellow crystals. They exhibit a vapor pressure of about 6 mbar at room temperature and can therefore be easily sublimated. The ^19^F NMR spectrum in C_6_D_6_/*n*‐pentane shows two singlets at −84.5 ppm (CF_3_) and −94.6 ppm (CF_2_), both with lead satellites. The ^3^ *J*(Pb,F) coupling constant of 23 Hz is rather large compared to mono‐, di‐, and triaryl(pentafluoroethyl)plumbanes (^3^ *J*(Pb,F) < 8 Hz). The same behavior can be observed for the ^2^ *J*(Pb,F) coupling constant (820 Hz) compared to ^2^ *J*(Pb,F) = 511 Hz for Pb(C_2_F_5_)_3_Tol. The ^207^Pb NMR spectrum in C_6_D_6_/*n*‐pentane shows a septet of broad multiplets at −245 ppm. Using the more polar solvent CD_3_CN, the signal is strongly shifted highfield to −547 ppm. The resonance of the CF_2_ group is also shifted toward higher field by about 4 ppm. This trend is also observed for Pb Me_3_Cl, Pb Et_3_Cl, Pb Ph_3_Cl, and other triorganylhalogenoplumbanes. The resonance of the central lead atom of PbEt_3_Cl is shifted from 472 (CHCl_3_) over 416 (CH_3_CN) to 305 ppm (pyridine) [31, 32, 33].

The IR spectrum of Pb(C_2_F_5_)_3_Cl (Figure 6) in the gas phase shows the characteristic vibrations of the PbCF_2_CF_3_ framework due to the absence of any other organyl groups. The band at highest energy (1330 cm^‑1^) is assigned to the symmetric and antisymmetric C─C stretching vibrations of the C_2_F_5_ groups. The C─F stretching vibration of the CF_3_ groups gives rise to a band at 1229 cm^‑1^. The bands at 1149 and 1105 cm^−1^ are assigned to the symmetric and antisymmetric C─F stretching vibrations of the CF_2_ groups. At 904 cm^−1^ the symmetric and antisymmetric Pb─C stretching vibrations are observed. The bending vibrations of the CF_2_ groups are found at 739 cm^‑1^. The Pb–Cl vibration is expected to be below 400 cm^−1^ and is therefore outside of the spectral range of the used spectrometer.

**FIGURE 6:**
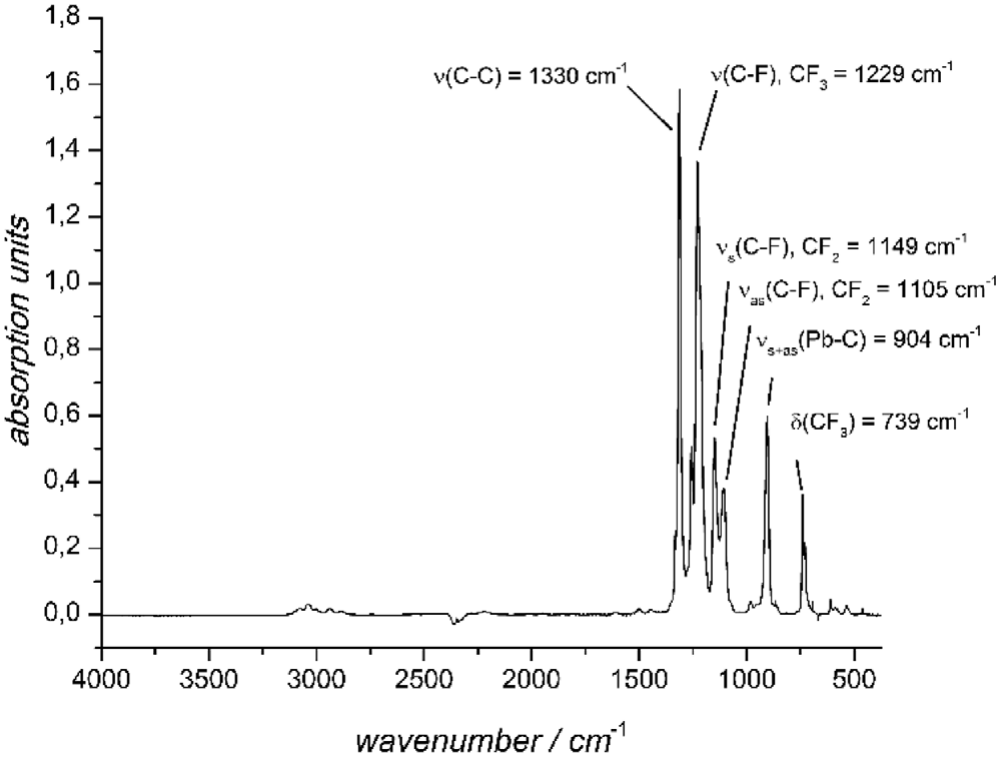
Gas phase IR spectrum of Pb(C_2_F_5_)_3_Cl with assignment of bands.

Pb(C_2_F_5_)_3_Cl is obtained as a crystalline solid and was studied by x‐ray structure analysis. It is located on the *C*_3_ axis in the trigonal space group *R3c*. The molecular structure in the solid state is shown in Figure 7. The three Cl─Pb1─C bond angles are exactly 90.0(1)° and the C1─Pb1─C1’ bond angles are exactly 120°. The coordination sphere is not tetrahedral as expected but is extended to a trigonal bipyramidal form. The Cl1─Pb1─Cl1’ and the Pb1─Cl1─Pb1’ bond angles of 180° and two nearly identical Pb─Cl distances lead to infinite linear chains of plumbane units in the solid state (see Figure 7, right side). The Pb1─Cl1 and Pb1─Cl1’ distances amount to 261.2 and 267.1 pm, respectively. The small difference of only 6 pm shows the high Lewis acidity of the lead atom. The comparison with PbPh_3_Cl [34], the only other chlorotriorganylplumbane structurally characterized in the solid state, underlines this. The Pb─Cl distances (270.6 and 294.7 pm) are clearly longer (9, respectively 28 pm) and differ by about 24 pm. Additionally, the chain structure is not linear but bent at the chlorine atoms with a Pb─Cl─Pb1 bond angle of 134.0°.

**FIGURE 7:**
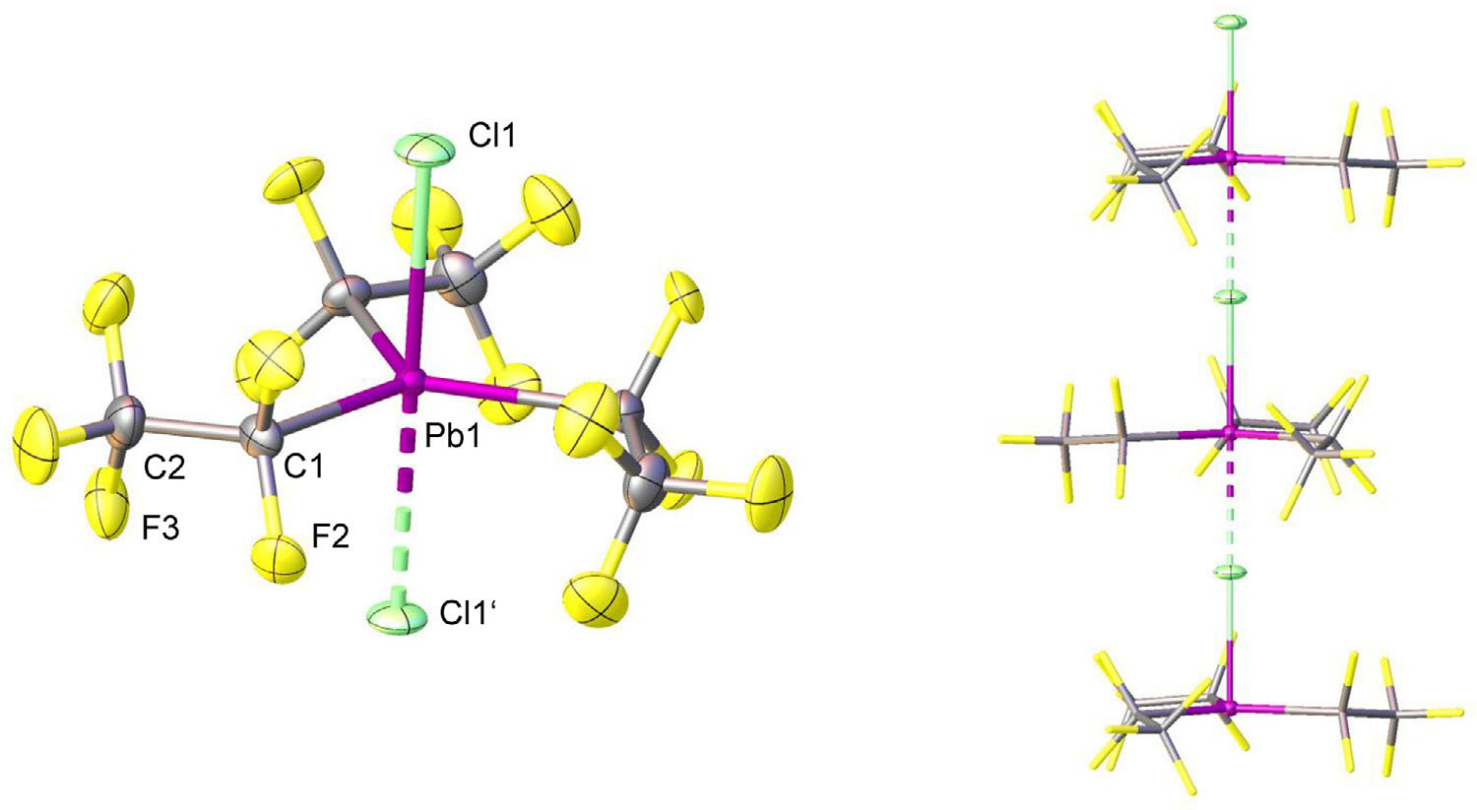
Molecular structure of Pb(C_2_F_5_)_3_Cl (left side, thermal ellipsoids shown at the 50% probability level); three units of the infinite chain structure (right side, C_2_F_5_ groups in wires/sticks model). Selected bond lengths [pm] and angles [°]: Pb1‐C1 228.8(4), Pb1‐Cl1 261.2(14), Pb1‐Cl1' 267.1(14), Cl1‐Pb1‐Cl1' 180.0, Pb1─Cl1─Pb1' 180.0, Cl1─Pb1─C1 90.0(1), C1”─Pb1─C1─C2 −6.1(2). Symmetry codes: ‘Y−X, Y, −1/2+Z’, "1−Y, 1+X−Y, +Z.

This also explains the shift of the lead atom in the ^207^Pb NMR spectra. In nonpolar solvents the structure is rather bridged, like in the solid state, and becomes more and more monomeric in polar solvents.

Besides the direct synthesis of the tetrakis(pentafluoroethyl)plumbane from Pb(OAc)_4_, Pb(C_2_F_5_)_4_ can also be synthesized from Pb(C_2_F_5_)_3_Cl with LiC_2_F_5_ (Scheme 8).

**SCHEME 8:**
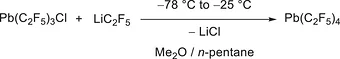
Synthesis of tetrakis(pentafluoroethyl)plumbane from chlorotris(pentafluoroethyl)plumbane.

In contrast to diarylbis(pentafluoroethyl)plumbanes, HBr is able to cleave the aryl group in Pb(C_2_F_5_)_3_Tol. The obtained bromotris(pentafluoroethyl)plumbane is not stable toward light and cannot be isolated selectively. When exposed to daylight it dimerizes and hexakis(pentafluoroethyl)diplumbane is obtained (Scheme 9); however, the bromine released in this reaction slowly decomposes the diplumbane.

**SCHEME 9:**
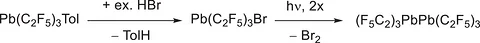
Synthesis of bromotris(pentafluoroethyl)plumbane from tolyltris(pentafluoroethyl)plumbane and dimerization to hexakis(pentafluoroethyl)diplumbane.

The reaction of Pb(C_2_F_5_)_3_Cl with SbF_3_ selectively affords the diplumbane (Scheme 10). To the best of our knowledge, hexakis(pentafluoroethyl)diplumbane is the first perfluorinated diplumbane. The yellow solid has a melting point just above room temperature and sublimates easily in vacuum by heating to 50°C.

**SCHEME 10:**
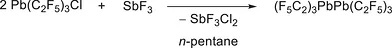
Synthesis of hexakis(pentafluoroethyl)diplumbane from chlorotris(pentafluoroethyl)plumbane with antimony trifluoride.

In the ^19^F NMR spectrum of Pb_2_(C_2_F_5_)_6_ the CF_3_ group is observed as a singlet at 82.6 ppm. The signal of the CF_2_ group is observed at 93.9 ppm with two sets of lead satellites (Figure 8).

**FIGURE 8:**
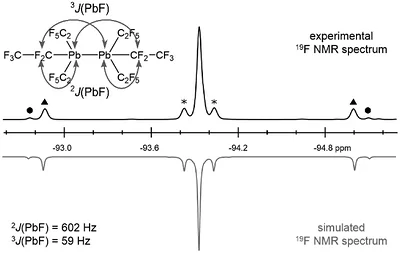
Experimental and simulated [27] CF_2_ region of the ^19^F NMR spectrum of Pb_2_(C_2_F_5_)_6_. Lead satellites (*, ▲) and higher order lead satellites are marked (●).

The two lead atoms in hexakis(pentafluoroethyl)diplumbane are chemically equivalent but magnetically inequivalent. This effect influences the signal of the CF_2_ group in the ^19^F NMR spectrum. One set of lead satellites arises due to the ^2^ *J*(Pb,F) coupling of 600 Hz, while the second set is caused by a ^3^ *J*(Pb,F) coupling of 59 Hz. In the isotopologue (C_2_F_5_)_3_^207^Pb^207^Pb(C_2_F_5_)_3_ the two lead atoms are magnetically inequivalent due to the long range lead fluorine coupling. These higher order lead satellites are shown in Figure 9, together with a simulation of the spectrum as a simplified A_6_A_6_’XX’ spin system.

**FIGURE 9:**
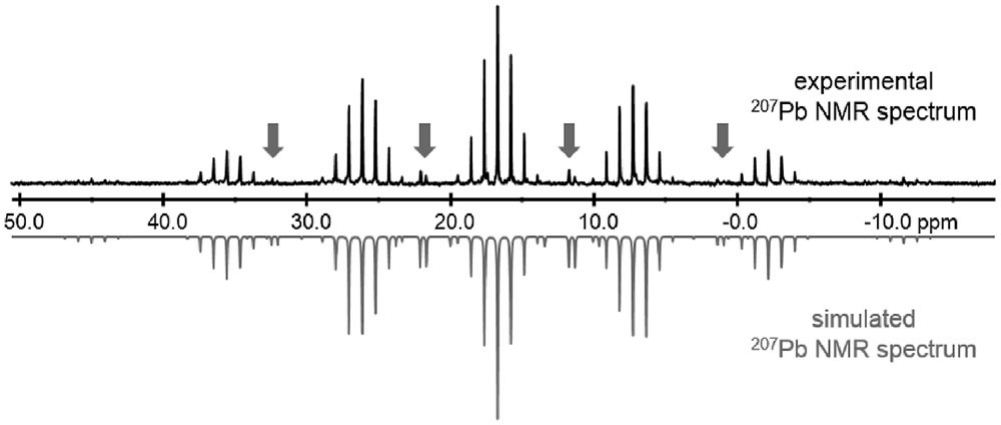
Experimental and simulated [34] ^207^Pb{^19^F} NMR spectrum of Pb_2_(C_2_F_5_)_6_. Only the fluorine atoms of the CF_3_ group are decoupled. Observable signals of the (C_2_F_5_)_3_^207^Pb^207^Pb(C_2_F_5_)_3_ isotopologue are marked. Simulation as an A_6_A_6_’XX’ spin system (^2^ *J*(Pb,F) = 600 Hz, ^3^ *J*(Pb,F) = 59 Hz, ^1^ *J*(Pb,Pb) = 5250 Hz).

Also, a lead–lead coupling over one bond has to be taken into account. ^1^ *J*(Pb,Pb) coupling constants vary over a wide range. Examples are Pb_2_Me_6_ [36] (+290 Hz) and Bu_3_PbPbEt_3_ which exhibits a ^1^ *J*(Pb,Pb) coupling constant 8126 Hz [33]. Because the isotopologue R_3_^207^Pb^207^PbR_3_ is present in an abundance of only 4% and its signal is superimposed by molecules containing a nonmagnetic lead isotope, the resonances are difficult to observe. In the fully coupled ^207^Pb NMR spectrum the resonance splits into a septet of septets of decets. To simplify the simulation the ^19^F decoupling is selectively employed on the CF_3_ group (Figure 9).

The absolute value for the ^1^ *J*(Pb,Pb) coupling was estimated to be 5250 Hz. The experiment and the simulation are in good agreement. The value of the ^1^ *J*(Pb,Pb) coupling constant agrees with the values reported for other diplumbanes.

Through slow sublimation of the compound a single crystal suitable for x‐ray structure analysis was obtained. Pb_2_(C_2_F_5_)_6_ crystallizes in the monoclinic space group *P*2_1_/*c* with *C_i_* symmetry, so the asymmetric unit consists of only one Pb(C_2_F_5_)_3_ unit (Figure 10).

**FIGURE 10:**
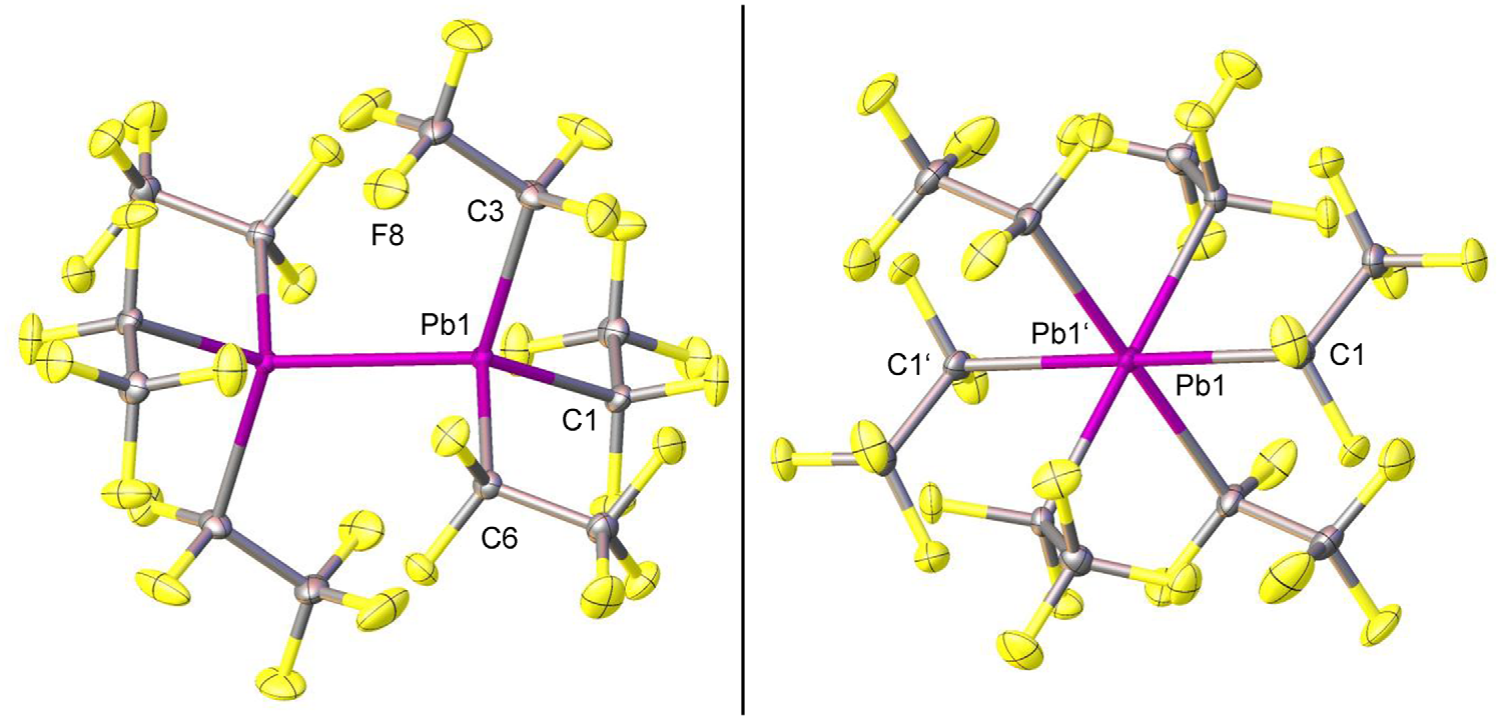
Molecular structure of Pb_2_(C_2_F_5_)_6_ in the solid state (thermal ellipsoids shown at the 50% probability level); right: view along the Pb─Pb bond. Selected bond lengths [pm] and angles [°]: Pb1─Pb1’ 286.116(18), Pb1C1 229.5(2), Pb1C3 229.7(3), Pb1C6 229.6(2), Pb1'─Pb1─C1 114.75(7), Pb1'─Pb1─C3 115.20(7), Pb1'─Pb1─C6 107.30(6), C1─Pb1─C3 105.63(9), C1─Pb1─C6 108.02(8), C3─Pb1─C6 105.38(9), C1─Pb1─Pb1’─C1’ 180. Symmetry codes for ‘: −X, −Y, −Z.

Hexakis(pentafluoroethyl)diplumbane is the first structurally characterized hexaalkyldiplumbane with electron‐withdrawing substituents. Other known diplumbanes mainly represent hexaaryldiplumbanes [27, 37, 38, 39, 40]. The coordination around the two lead centers is basically tetrahedral, with slightly increased Pb─Pb─C bond angles and slightly decreased C─Pb1─C bond angles. The C_2_F_5_ groups are in a *gauche* conformation, which is also found in literature‐known hexaorganyldiplumbanes. Due to the inversion center, the C1Pb1Pb1’C1’ torsion angle is exactly 180°.

The Pb─Pb bond length of 286.1 pm does not differ from other diplumbanes (Table 3). The Pb─Pb bond increases with a larger steric demand of the substituents. The Pb─C bond lengths of Pb_2_(C_2_F_5_)_6_ (229.6 pm) are the longest compared to the non‐fluorinated diplumbanes (219.8 to 227 pm). This is addressed as the CH_3_/CF_3_ substitution effect by Oberhammer and is due to small repulsive electrostatic interactions in the E─C_F_ bond [41]. The conformation between two vicinal carbons is mostly staggered with angles close to the ideal 60°, with the exception of Pb_2_(*p*‐Tol)_6_ and Pb_2_(*c*‐Hex)_6_.

**TABLE 3:** Comparison of Pb─Pb and Pb─C bond lengths [pm] and torsion angles [°] of selected hexaorganylplumbanes.

	*d*(Pb–Pb)	*d*(Pb–C)	C–Pb–Pb’–C’^a^
Pb_2_Ph_6_ [37]	284.4	222.2^c^	57.5
*Pb_2_* (*p*‐Tol)_6_ [27]	285.1	219.8^c^	30.3
Pb_2_(C_2_F_5_)_6_	286.1	229.6^c^	60.0
Pb_2_(*c*‐Hex)_6_ [38]	287.6	227^c^	34.6
Pb_2_(*o*‐Tol)_6_ [39]	289.5	224.5^c^	59.9
Pb_2_(*t*‐Bu)_6_ [39]	293.7	n.a.	n.a.
Pb_2_[Me_2_{C(SiMe_3_)_3_}]_6_ [40]	296.8	223.8^b^, ^c^	59.9

^a^ Smallest angle.

^b^ Methyl groups only.

^c^ Mean value.

The color of these compounds changes from colorless (Pb_2_Ph_6_) over light yellow (Pb_2_(*p*‐Tol)_6_) and intense yellow (Pb_2_(C_2_F_5_)_6,_ Pb_2_(*c*‐Hex)_6_) to orange/yellow (Pb_2_[Me_2_{C(SiMe_3_)_3_}]_6_). The color seems to be related to length of the Pb─Pb bond. A strong stretching or a radical character is assumed in the literature [38]. It is also reported that all these compounds are sensitive toward light.

The reaction of Pb(C_2_F_5_)_4_ with iodide or bromide salts affords the tris(pentafluoroethyl)plumbate(II) anion, [Pb(C_2_F_5_)_3_]^−^ (Scheme 11).

**SCHEME 11:**
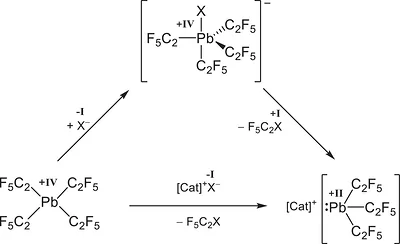
Synthesis of the tris(pentafluoroethyl)plumbate(II) anion, [Pb(C_2_F_5_)_3_]^−^, from Pb(C_2_F_5_)_4_ with halides (cat = PPh_4_, N(*n*‐Bu)_4_; X = Br, I).

The reaction is much faster for iodide than for bromide. It is assumed that the reaction proceeds over a pentacoordinated transition state and that in the second step a reductive elimination takes place. The generation of C_2_F_5_Br or C_2_F_5_I is clearly proven by ^19^F NMR spectroscopy. The pentacoordinated intermediate can also be observed by NMR spectroscopy using bromide salts; for iodide salts the reaction is too fast to be monitored. Chloride forms a stable adduct with Pb(C_2_F_5_)_4_ in solution; no C_2_F_5_Cl can be observed. A large highfield shift for the CF_2_ groups can be observed. For the chlorine adduct the CF_2_ group shifts from −94.0 (free Pb(C_2_F_5_)_4_) to −103.5 ppm.

In this reaction, iodide and even bromide are oxidized from I to +I and in the same step lead is reduced to +II. For the oxidation of chlorine, the oxidation potential of lead(IV) is not high enough. Lead(IV) salts are used as potential oxidation agents since 1930, for example for the glycol cleavage with lead tetraacetate [42].

Due to the still inefficient synthesis of Pb(C_2_F_5_)_4_, however, it would be much more attractive to use Pb(C_2_F_5_)_3_Cl as a starting material. Patorra [43] showed that the reaction of Pb(CF_3_)_3_Br with tertiary phosphanes (PR_3_, R = Me, Ph, NEt_2_) yields the tris(trifluoromethyl)plumbate(II), which decomposes above 40°C. The reaction of Pb(C_2_F_5_)_3_Cl with PR_3_ (R = Me, Ph) furnishes [PR_3_Cl][Pb(C_2_F_5_)_3_] as a room temperature stable compound, which again highlights the increased stability of pentafluoroethyl substituted compounds compared to trifluoromethyl compounds [12]. The NMR spectroscopic properties differ a lot from the discussed lead(IV) compounds (Table 4).

**TABLE 4:** NMR spectroscopic parameters of [PPh_3_Cl][Pb(C_2_F_5_)_3_], [Pb(CF_3_)_3_]^−^ [43], and Li[PbPh_3_] [44].

	[Pb(C_2_F_5_)_3_][^a^]	[Pb(CF_3_)_3_][^b^]	Li[PbPh_3_][^b^]
*δ*(^207^Pb)/ppm	1608	1228	1062
*δ*(^19^F, CF_3_)/ppm	−82.9	−36.5	‐/‐
*δ*(^19^F, CF_2_)/ppm	−113.8	‐/‐	‐/‐
^2^ *J*(Pb,F)/Hz	42	−205	‐/‐
^3^ *J*(Pb,F)/Hz	61	‐/‐	‐/‐
^1^ *J*(Pb,C)/Hz	1390	−1682	1030
^2^ *J*(Pb,C)/Hz	29	‐/‐	59
^3^ *J*(F,F)/Hz	2	‐/‐	‐/‐
*^n^J*(F,F)/Hz	2 (^5^ *J*)	3 (^4^ *J*)	‐/‐

^a^ CD_2_Cl_2._

^b^ THF.

Characteristic for anionic lead(II) compounds is a distinct lowfield shift of the lead atom in the ^207^Pb NMR spectrum compared to lead(IV) [33]. While the resonance of Pb(C_2_F_5_)_4_ in the ^207^Pb NMR spectrum is observed at *δ* = −420 ppm, it is shifted to 1608 ppm for [Pb(C_2_F_5_)_3_]^−^. The ^1^ *J*(Pb,C) coupling constant is exceptionally large, which agrees with literature values.

While the ^2^ *J*(Pb,F) coupling constant decreases by several hundreds of Hertz, the ^3^ *J*(Pb,F) coupling constant, which usually is barely observable, now even exceeds the ^2^ *J*(Pb,F) coupling constant. The ^207^Pb NMR spectrum shows therefore a decet of septets (Figure 11).

**FIGURE 11:**
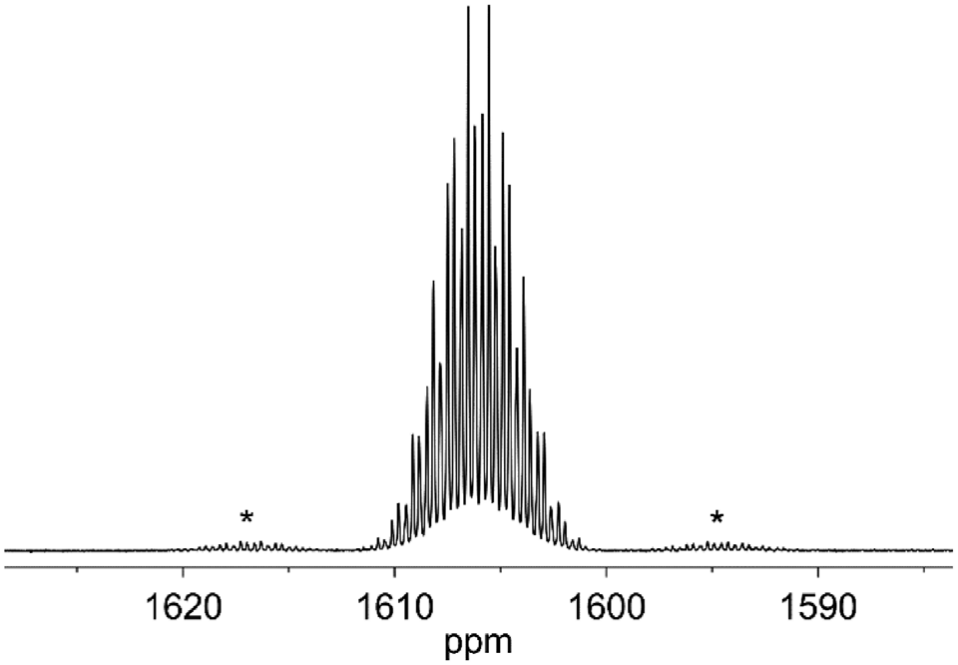
^207^Pb NMR spectrum of [PPh_3_Cl][Pb(C_2_F_5_)_3_] in CD_2_Cl_2_. Due to coupling to nine, respectively six equivalent fluorine atoms a decet of septets is observed. ^13^C satellites (^1^ *J*(Pb,C) = 1390 Hz) (*).

The ^19^F NMR spectrum is dominated by two multiplets (Figure 12). Due to short and long range couplings, ^3^ *J*(F,F) ≙ ^5^ *J*(F,F) = 2 Hz, a triplet of quintets for the CF_3_ group and a quartet of septets for the CF_2_ group is observed.

**FIGURE 12:**
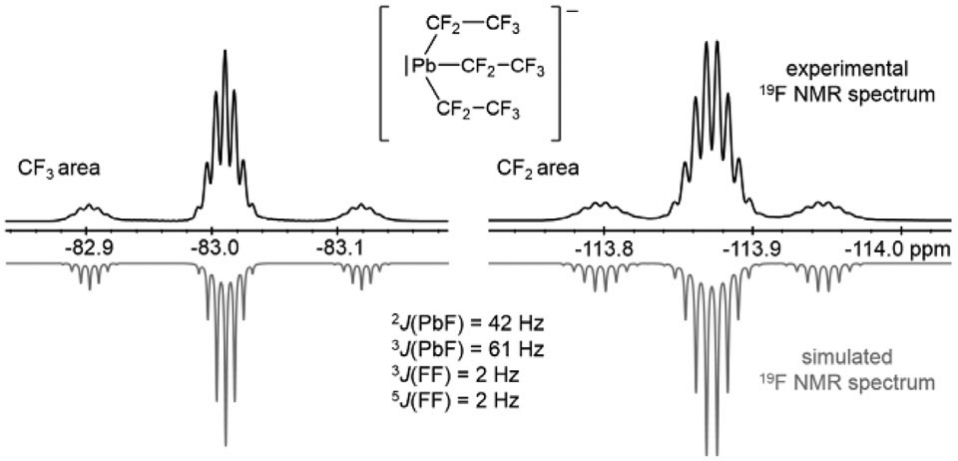
Experimental and simulated [35] CF_3_ and CF_2_ region of the ^19^F NMR spectrum of [PPh_3_Cl][Pb(C_2_F_5_)_3_] in CD_2_Cl_2_. Simulation as an 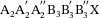 spin system (A = CF_2_, B = CF_3_, X = Pb), both signals scaled to the same height.

The formation of the chlorophosphonium ion is impressively shown in the ^31^P{^1^H} NMR spectrum. The ^31^P chemical shift of 66 ppm is in agreement with literature references [45, 46]. The spectrum reveals the ^35^Cl/^37^Cl isotope shift (Figure 13). In fluorine chemistry, the chlorine isotope shift is well known. The most common reference substance for ^19^F NMR spectra is the resonance of CF^35^Cl_2_^37^Cl, the most abundant isotopologue of CFCl_3_ [47, 48]. The ratio of the ^35^Cl/^37^Cl isotopologues is 74.5/24.5 [20], which can be clearly observed in the ^31^P{^1^H} NMR spectrum of [PPh_3_Cl][Pb(C_2_F_5_)_3_].

**FIGURE 13:**
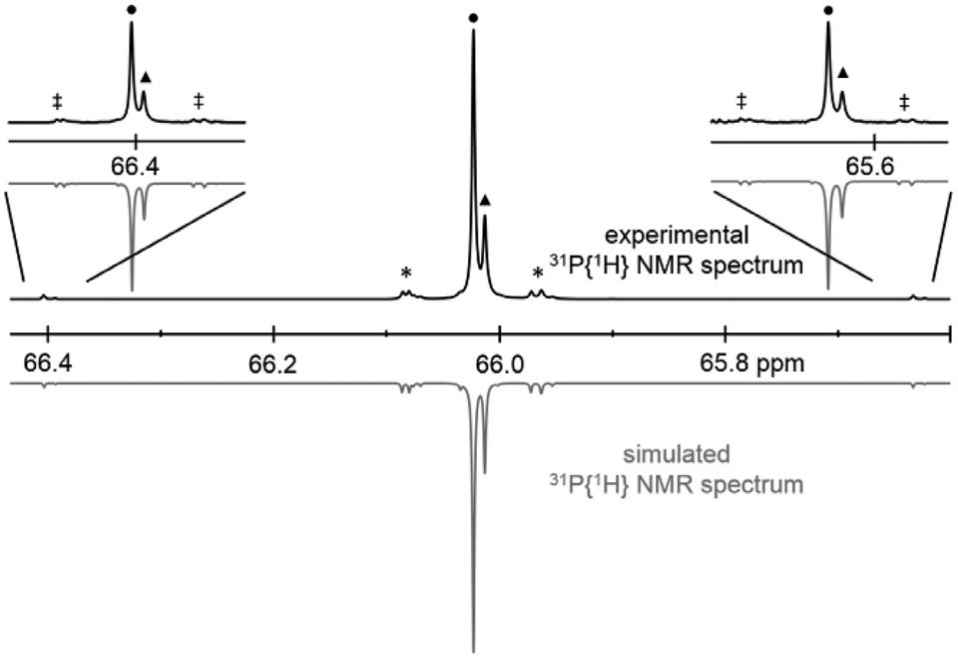
Experimental and simulated [35] ^31^P{^1^H} NMR spectrum of [PPh_3_Cl][Pb(C_2_F_5_)_3_] in CD_2_Cl_2_. Simulation as an $A_{3}B_{6}C_{6}D_{3}\mathrm{XY}$ spin system (A = B = C = D = C_arom_, X = P, Y = Cl). Expansion of the outermost ^13^C satellites (^1^ *J*(P,C)). Signals arising from the isotopologue with ^35^Cl, respectively ^37^Cl are marked (● and ▲), overlapping ^13^C satellites (*; ^2^ *J*(P,C), ^3^ *J*(P,C)) and ^13^C satellites on the ^13^C satellites (‡).

The isotope shift of 1.23 Hz was measured at 121.5 MHz and 25°C. This value is about half the amount of the value for PCl_3_ (3.1 Hz, 162 Mhz, ca. −100°C) [49], which can be explained by the temperature‐dependency of the isotope shift [50].

By slow diffusion of *n*‐pentane into a solution of [PPh_3_Cl][Pb(C_2_F_5_)_3_] in CH_2_Cl_2_ a single crystal suitable for x‐ray structure analysis was obtained. [PPh_3_Cl][Pb(C_2_F_5_)_3_] crystallizes in the orthorhombic space group *P*2_1_2_1_2_1_ (Figure 14). The crystal structure of [Pb(C_2_F_5_)_3_]^−^ represents the first plumbate(II) anion with fluorinated groups. The lead atom of the plumbate anion is in a pseudo‐trigonal pyramidal and the phosphorus atom of the phosphonium cation is in a tetrahedral coordination sphere. The structure of the cation is consistent with the literature [51]. The Pb─C bond lengths are all close to 239 pm and are longer than for [K{[18]crown‐6}][PbPh_3_] [52] (232 to 234 pm) or [Li(pmdeta)][PbPh_3_] [53] (228 to 230 pm). As for [Pb(C_2_F_5_)_3_]^−^, this can be rationalized by the effects described by Oberhammer [41]. The CPbC bond angles (85.0°–96.2°) are smaller compared to those of [K{[18]crown‐6}][PbPh_3_] (93.3–96.9).

**FIGURE 14:**
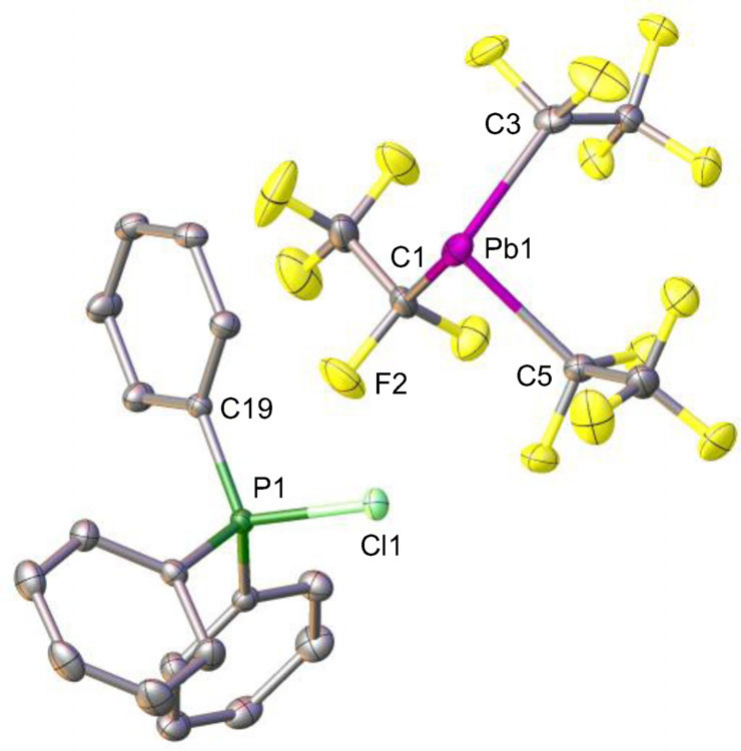
Molecular structure of [PPh_3_Cl][Pb(C_2_F_5_)_3_] (thermal ellipsoids are shown at the 50% probability level, hydrogen atoms omitted for clarity). Selected bond lengths [pm] and angles [°]: Pb1C1 239.4(3), Pb1C3 239.0(3), Pb1C5 238.6(3), P1Cl1 199.01(9), Pb1Cl 436.41(7), C1Pb1C3 96.25(10), C1Pb1C5 85.00(11), C3Pb1C5 95.37(10), Pb1─C1─C2 117.5(2), Pb1─C3─C4 129.2(2), Pb1─C5─C6 114.3(2).

This indicates the absence of a hybridization between the 6*s* and the 6*p* orbitals and shows the high *s* character of the lead lone pair of [Pb(C_2_F_5_)_3_]^−^, which is consistent with Bent's rule [54]. This was also observed for the isoelectronic and isostructural Bi(C_2_F_5_)_3_ [55]. The bismuthane also shows a trigonal pyramidal coordination and C─Bi─C angles close to 90°. Additionally, the lead lone pair is very unreactive. The plumbate anion can only be methylated with methyl triflate; no reaction can be observed with methyl iodide or allyl bromide.

In the solid state, no Pb–Cl contacts are observed. The lead chlorine distance of 436 pm exceeds the sum of the van der Waals radii (380 pm) [20].

## 3 Conclusion

In this work we reported the synthesis of tetrakis(pentafluoroethyl)plumbane along with the solid state structure of the 1,10‐phenanthroline adduct. Furthermore, the synthesis of functionalized tris(pentafluoroethyl)plumbanes is shown. The synthesis employed the use of aryl substituents as protective groups and yielded a series of pentafluoroethyl lead compounds with high yields. Aryl protecting groups can be selectively removed by use of gaseous HCl. The resulting plumbanes, Pb(C_2_F_5_)*_n_* Ar_4‐_ *_n_* (*n* = 1 – 3), were extensively studied by NMR spectroscopy and x‐ray crystallography. The reaction of Pb(C_2_F_5_)_3_Tol with HCl selectively leads to Pb(C_2_F_5_)_3_Cl, which showed a pentacoordinated lead atom in the solid state. The reaction with SbF_3_ furnishes the hexakis(pentafluoroethyl)diplumbane, a stable lead(III) compound. By reaction of the chloroplumbane with PPh_3_ a phosphonium plumbate is obtained. The molecular structure of [PPh_3_Cl][Pb(C_2_F_5_)_3_] in the solid states shows the high *s* character of the lead lone pair.

## Conflicts of Interest

The authors declare no conflicts of interest.

## Supporting information



The NMR spectra of all compounds (Figures S1–S73) and the x‐ray diffraction data (Tables S1 and S2) are provided in the Supporting Information File (DOC). The authors have cited additional references within the Supporting Information [57, 58].
**Supporting File 1**: chem71402‐sup‐0001‐SuppMat.docx

## Data Availability

The data that supports the findings of this study are available in the Supporting Information of this article.

## References

[1] D. Seyferth , “The Rise and Fall of Tetraethyllead. 1. Discovery and Slow Development in European Universities, 1853−1920,” Organometallics 22, no. 12 (2003): 2346–2357, 10.1021/om030245v.

[2] D. Seyferth , “The Rise and Fall of Tetraethyllead. 2,” Organometallics 22, no. 25 (2003): 5154–5178, 10.1021/om030621b.

[3] J. Burschka , N. Pellet , S.‐J. Moon , et al., “Sequential Deposition as a Route to High‐Performance Perovskite‐Sensitized Solar Cells,” Nature 499, no. 7458 (2013): 316–319, 10.1038/nature12340. 23842493

[4] R. Eujen and A. Patorra , “Trifluormethylsubstituierte Plumbane. Synthese, Eigenschaften und NMR‐Spektren der verbindungen (CF_3_)nPbR_3_‐n (*n* = 1–3) und (C),” Journal of Organometallic Chemistry 438, no. 1‐2 (1992): 57–75, 10.1016/0022-328X(92)88006-5.

[5] R. Eujen and A. Patorra , “Trifluormethyl‐substituierte Plumbane—Darstellung, Eigenschaften und NMR‐spektroskopische Charakterisierung von CF_3_PbR_2_H (R = CH_3_, C_2_H_5_) sowie von CF_3_Pb(II)‐Derivaten,” Journal of Organometallic Chemistry 481, no. 1 (1994): 75–82, 10.1016/0022-328X(94)85011-9.

[6] T. J. Juhlke , J. I. Glanz , and R. J. Lagow , “The Synthesis of Tetrakis(trifluoromethyl)Lead,” Inorganic Chemistry 28, no. 5 (1989): 980–981, 10.1021/ic00304a038.

[7] H. D. Kaesz , J. R. Phillips , and F. G. A. Stone , Chemistry & Industry (1959): 1409–1410.

[8] H. D. Kaesz , J. R. Phillips , and F. G. A. Stone , “Preparation and Study of Some Perfluoroalkyl Compounds of Tin and Lead 1,2,” Journal of the American Chemical Society 82, no. 24 (1960): 6228–6232, 10.1021/ja01509a004.

[9] H. Lange and D. Naumann , “Perfluororganocadmium‐verbindungen als sehr wirksame perfluoralkylierungs‐ und perfluorphenylierungsmittel zur darstellung von perfluororganozinn‐ und ‐blei‐verbindungen [1],” Journal of Fluorine Chemistry 27, no. 3 (1985): 309–318, 10.1016/S0022-1139(00)81311-1.

[10] H. Beckers , (Bergische Universität—Gesamthochschule Wuppertal, 1987).

[11] H. Beckers , H. Bürger , and R. Eujen , “Dialkylamino‐Trifluormethylsilane,” Zeitschrift für anorganische und allgemeine Chemie 563, no. 1 (1988): 38–47, 10.1002/zaac.19885630106.

[12] S. Steinhauer , J. Bader , H.‐G. Stammler , N. Ignat′Ev , and B. Hoge , “Synthese von Tris‐ und Tetrakis(pentafluorethyl)silanen,” Angewandte Chemie 126, no. 20 (2014): 5307–5310, 10.1002/ange.201400291, Angew. Chem. Int. Ed., 2014, 53, 5206–5209. 24692241

[13] M. H. Königsmann , (Universität Bremen, 2005).

[14] M. Henrich , A. Marhold , A. Kolomeitsev , A. Kadyrov , G.‐V. Röschenthaler , and J. Barten , DE 101 (2002), 28 703 A 1.

[15] B. Waerder , S. Steinhauer , B. Neumann , et al., “Festkörperstruktur eines Li/F‐Carbenoids: Pentafluorethyllithium,” Angewandte Chemie 126, no. 43 (2014): 11824–11828, 10.1002/ange.201406564, Angew. Chem. Int. Ed., 2014, 53, 11640–11644. 25205058

[16] S. Pelzer , B. Neumann , H.‐G. Stammler , N. Ignat'ev , and B. Hoge , “Synthesis of Tris(pentafluoroethyl)germanes,” Chemistry—A European Journal 22, no. 10 (2016): 3327–3332, 10.1002/chem.201504626. 26797789

[17] J. Klösener , (Universität Bielefeld, 2013).

[18] S. Solyntjes , J. Bader , B. Neumann , H.‐G. Stammler , N. Ignat'ev , and B. Hoge , “Pentafluoroethyl Bismuth Compounds,” Chemistry—A European Journal 23 (2016): 1557–1567, 10.1002/chem.201604910. 27862439

[19] Deposition numbers 2533633–2533639 contain the supplementary crystallographic data for this paper. These data are provided free of charge by the joint Cambridge Crystallographic Data Centre and Fachinformationszentrum Karlsruhe Access Structures service.

[20] A. F. Holleman , E. Wiberg , and N. Wiberg , Lehrbuch der Anorganischen Chemie (de Gruyter, 2007), 10.1515/9783110177701.

[21] T. M. Klapötke , B. Krumm , M. Niemitz , K. Polborn , and C. M. Rienäcker , “Tetrakis(pentafluorophenyl)lead(IV): Crystal Structure, Semiempirical PM3 Calculations and NMR Studies,” Journal of Fluorine Chemistry 104, no. 2 (2000): 129–133, 10.1016/S0022-1139(00)00210-4.

[22] H. Gilman , L. Summers , and R. W. Leeper , “The Reaction of Aryllithium Compounds With Lead Dichloride. Triphenylleadlithium,” Journal of Organic Chemistry 17, no. 4 (1952): 630–640, 10.1021/jo01138a018.

[23] H. Gilman and J. D. Robinson , “The Preparation of Triphenyl Lead Chloride and Diphenyl Lead Dichloride,” Journal of the American Chemical Society 51, no. 10 (1929): 3112–3114, 10.1021/ja01385a033.

[24] V. Busetti , M. Mammi , A. Signor , and A. Del Pra , “A Refinement of the Crystal Structure of Tetraphenyl‐Lead,” Inorganica Chimica Acta 1 (1967): 424–428, 10.1016/S0020-1693(00)93216-X.

[25] H. Preut and F. Huber , “Redetermination of Tetraphenyllead,” Acta Crystallographica Section C Crystal Structure Communications 49, no. 7 (1993): 1372–1373, 10.1107/S0108270193001878.

[26] K. Claborn , B. Kahr , and W. Kaminsky , “Calculations of Optical Properties of the Tetraphenyl‐X Family of Isomorphous Crystals (X = C, Si, Ge, Sn, Pb),” CrystEngComm 4, no. 46 (2002): 252–256, 10.1039/B202304K.

[27] C. Schneider and M. Dräger , “Über Gemischte Gruppe 14—Gruppe 14‐Bindungen,” Journal of Organometallic Chemistry 415, no. 3 (1991): 349–362, 10.1016/0022-328X(91)80135-7.

[28] C. Schneider‐Koglin , B. Mathiasch , and M. Dräger , “Über Tetraaryl‐Methan‐Analoga in Der Gruppe 14,” Journal of Organometallic Chemistry 469, no. 1 (1994): 25–32, 10.1016/0022-328X(94)80074-X.

[29] S. Pelzer , B. Neumann , H.‐G. Stammler , N. Ignat'ev , and B. Hoge , “Synthesis of Bis(pentafluoroethyl)germanes,” Chemistry—A European Journal 22, no. 14 (2016): 4758–4763, 10.1002/chem.201505084. 26880212

[30] B. Wrackmeyer , in Annual Reports on NMR Spectroscopy, eds. G. A. Webb (Academic Press, 2002).

[31] E. Capek and K. E. Schwarzhans , “ ^207^Pb‐NMR‐Untersuchungen an Bleiorganylen,” Monatshefte für Chemie—Chemical Monthly 118, no. 4 (1987): 419–426, 10.1007/BF00809924.

[32] T. N. Mitchell , J. Gmehling , and F. Huber , Journal of the Chemical Society 8 (1978): 960–964.

[33] B. Wrackmeyer and K. Horchler in Annual Reports on NMR Spectroscopy, eds. G. A. Webb (Academic Press, 1983).

[34] H. Preut and F. Huber , “Die Kristall‐ und Molekülstrukturen von Triphenylbleichlorid und Triphenylbleibromid,” Zeitschrift für anorganische und allgemeine Chemie 435, no. 1 (1977): 234–242, 10.1002/zaac.19774350131.

[35] P. H. M. Budzelaar , gNMR Version 50; IvorySoft (2006).

[36] J. D. Kennedy and W. McFarlane , “The Magnitude and Sign of ^1^J(^207^Pb‐^207^Pb) in Hexamethyldilead,” Journal of Organometallic Chemistry 80, no. 3 (1974): C47–C49, 10.1016/S0022-328X(00)80021-2.

[37] H. Preut and F. Huber , “Die Kristall‐ und Molekülstruktur des Hexaphenyldiplumbans,” Zeitschrift für anorganische und allgemeine Chemie 419, no. 1 (1976): 92–96, 10.1002/zaac.19764190115.

[38] N. Kleiner and M. Drager , “Über gemischte Bindungen in der IV. Hauptgruppe, II [1]. Hexacyclohexylethan‐Analoga Pb_2_(c‐Hex)_6_, (c‐Hex)_3_ Pb–Sn(c‐Hex)_3_ und Sn_2_(c‐Hex)_6_/On Mixed Group IVb—Group IVb Bonds, II [1]. Hexacyclohexylethane Analogues Pb_2_ (c‐Hex)_6_, (c‐Hex)_3_ Pb–Sn(c‐Hex)_3_, and Sn_2_ (c‐Hex)_6_ ,” Zeitschrift für Naturforschung B 40b (1985): 477–483, 10.1515/znb-1985-0407.

[39] C. Schneider‐Koglin , K. Behrends , and M. Dräger , “Über Gemischte Gruppe 14‐Gruppe 14‐Bindungen,” Journal of Organometallic Chemistry 448, no. 1‐2 (1993): 29–38, 10.1016/0022-328X(93)80063-H.

[40] S. M. Whittaker , F. Cervantes‐Lee , and K. H. Pannell , “Synthesis, Single‐Crystal X‐Ray Structural Characterization, and Reactivity of a Hexaalkyldiplumbane, TsiMe_2_Pb‐PbMe_2_Tsi, Tsi = (Me_3_Si)_3_C,” Inorganic Chemistry 33, no. 26 (1994): 6406–6408, 10.1021/ic00104a061.

[41] H. Oberhammer , “On the Structural Effects of CF_3_ Groups,” Journal of Fluorine Chemistry 23, no. 2 (1983): 147–162, 10.1016/S0022-1139(00)84976-3.

[42] R. Criegee , “Eine oxydative Spaltung von Glykolen (II. Mitteil. über Oxydationen mit Blei(IV)‐salzen),” Berichte der deutschen chemischen Gesellschaft (A and B Series) 64, no. 2 (1931): 260–266, 10.1002/cber.19310640212.

[43] A. Patorra , (Bergische Universität—Gesamthochschule Wuppertal, 1994).

[44] U. Edlund , T. Lejon , P. Pyykko , T. K. Venkatachalam , and E. Buncel , “Lithium‐7, Silicon‐29, Tin‐119, and Lead‐207 NMR Studies of Phenyl‐substituted Group 4 Anions,” Journal of the American Chemical Society 109, no. 20 (1987): 5982–5985, 10.1021/ja00254a015.

[45] E. Krawczyk , A. Skowrońska , and J. Michalski , Journal of the Chemical Society 23 (2002): 4471–4478.

[46] S. J. Hwang , D. C. Powers , A. G. Maher , and D. G. Nocera , “Tandem Redox Mediator/Ni(ii) Trihalide Complex Photocycle for Hydrogen Evolution From HCl,” Chemical Science 6, no. 2 (2015): 917–922, 10.1039/C4SC02357A. PMC5811117 29560177

[47] J. W. Emsley and L. Phillips , “Fluorine Chemical Shifts,” Progress in Nuclear Magnetic Resonance Spectroscopy 7 (1971): 1–520, 10.1016/0079-6565(71)90002-1.

[48] P. R. Carey , H. W. Kroto , and M. A. Turpin , “Chlorine Isotope Effects on the ^19^F Resonance of CCl_3_F and the Use of This Signal as a Reference in High‐Resolution Nuclear Magnetic Resonance,” Journal of the Chemical Society D: Chemical Communications 5 (1969): 188, 10.1039/c29690000188.

[49] M. J. Buckingham , G. E. Hawkes , I. M. Ismail , and P. J. Sadler , Journal of the Chemical Society 6 (1982): 1167.

[50] M. Tordeux , C. Wakselman , O. Jarjayes , and C. G. Béguin , “ ^37^Cl/^35^Cl and ^13^C/^12^C Isotope Effects on the Fluorine Shielding in Several Two‐Carbon Chlorofluorocarbons,” Magnetic Resonance in Chemistry 39, no. 6 (2001): 301–310, 10.1002/mrc.846.

[51] J. H. Enemark and J. A. Ibers , “Crystal and Molecular Structure of Chlorotriphenylphosphonium Bis(Cis‐1,2‐Bis(trifluoromethyl)ethene‐1,2‐Dithiolato)Gold, [PCl(C_6_H_5_)_3_][Au(S_2_C_2_(CF_3_)_2_)_2_],” Inorganic Chemistry 7, no. 12 (1968): 2636–2642, 10.1021/ic50070a035.

[52] C. Kleeberg , “On the Structural Diversity of [K(18‐crown‐6)EPh_3_] Complexes (E = C, Si, Ge, Sn, Pb): Synthesis, Crystal Structures and NOESY NMR Study,” Dalton Transactions 42, no. 23 (2013): 8276, 10.1039/c3dt50523e. 23599065

[53] D. R. Armstrong , M. G. Davidson , D. Moncrieff , D. Stalke , and D. S. Wright , “The Observation of a Pb–Li Bond; Synthesis, Structure and Model Molecular Orbital (MO) Calculations on the Monomeric Ph_3_ Pb–Li·(Pmdeta) Complex [pmdeta = (Me_2_NCH_2_CH_2_)_2_NMe],” Journal of the Chemical Society, Chemical Communications 19 (1992): 1413–1415, 10.1039/C39920001413.

[54] H. A. Bent , “An Appraisal of Valence‐Bond Structures and Hybridization in Compounds of the First‐Row Elements,” Chemical Reviews 61, no. 3 (1961): 275–311, 10.1021/cr60211a005.

[55] S. Solyntjes (Universität Bielefeld, 2015).

