# Supplementary material for: Syntheses and Properties of Pentafluoroethylplumbanes in the Oxidation States +II, +III, and +IV
Source: Chemistry. 2026 Jul 18;32(39):e71402. doi: 10.1002/chem.71402 (PMC13715457; doi:10.1002/chem.71402)
Supplement: Supplementary file 1 [file CHEM-32-e71402-s001.docx]

**Supporting Information**

Syntheses and properties of pentafluoroethylplumbanes in the oxidation state +II, +III and +IV

Michael Grasse^a^, Lukas Hartmann^a^, Beate Neumann^a^, Hans-Georg Stammler^a^, Berthold Hoge^a^*

^a^ Centrum für Molekulare Materialien, Fakultät für Chemie, Universität Bielefeld, Universitätsstraße 25, 33615 Bielefeld, Germany

* Email: b.hoge@uni-bielefeld.de

**Table of Contents**

[**1 General**	4](#_Toc224126053)

[**2 Synthetic procedures**	5](#_Toc224126054)

[**2.1** Synthesis of tetrakis(pentafluoroethyl)plumbane	5](#_Toc224126055)

[**2.2** Synthesis of tetrakis(pentafluorethyl)-1,10-phenantrolinelead(IV)	6](#_Toc224126056)

[**2.3** Synthesis of tetraphenylplumbane	7](#_Toc224126057)

[**2.4** Synthesis of tetratolylplumbane	8](#_Toc224126058)

[**2.5** Synthesis of tetraanisylplumbane	9](#_Toc224126059)

[**2.6** Synthesis of bis(pentafluoroethyl)diphenylplumbane	10](#_Toc224126060)

[**2.7** Synthesis of bis(pentafluoroethyl)ditolylplumbane	11](#_Toc224126061)

[**2.8** Synthesis of bis(pentafluoroethyl)dianisylplumbane	12](#_Toc224126062)

[**2.9** Synthesis of (pentafluoroethyl)triphenylplumbane	13](#_Toc224126063)

[**2.10** Synthesis of (pentafluoroethyl)tritolylplumbane	15](#_Toc224126064)

[**2.11** Synthesis of tris(pentafluoroethyl)phenylplumbane	16](#_Toc224126065)

[**2.12** Synthesis of tris(pentafluoroethyl)tolylplumbane	18](#_Toc224126066)

[**2.13** Synthesis of tris(pentafluoroethyl)anisylplumbane	20](#_Toc224126067)

[**2.14** Synthesis of chlorotris(pentafluoroethyl)plumbane	21](#_Toc224126068)

[**2.15** Synthesis of hexakis(pentafluorethyl)diplumbane	22](#_Toc224126069)

[**2.16** Synthesis of chlorotriphenylphosphonium-tris(pentafluoroethyl)plumbate	23](#_Toc224126070)

[**3 NMR spectra**	24](#_Toc224126071)

[**3.1** NMR spectra of tetrakis(pentafluoroethyl)plumbane	24](#_Toc224126072)

[**3.2** NMR spectra of tetrakis(pentafluoroethyl)-1,10-phenantrolinelead(IV)	26](#_Toc224126073)

[**3.3** NMR spectra of tetraphenylplumbane	28](#_Toc224126074)

[**3.4** NMR spectra of tetratolylplumbane	29](#_Toc224126075)

[**3.5** NMR spectra of tetraanisylplumbane	30](#_Toc224126076)

[**3.6** NMR spectra of bis(pentafluoroethyl)diphenylplumbane	32](#_Toc224126077)

[**3.7** NMR spectra of bis(pentafluoroethyl)ditolylplumbane	34](#_Toc224126078)

[**3.8** NMR spectra of bis(pentafluoroethyl)dianisylplumbane	37](#_Toc224126079)

[**3.9** NMR spectra of (pentafluoroethyl)triphenylplumbane	40](#_Toc224126080)

[**3.10** NMR spectra of (pentafluoroethyl)tritolylplumbane	42](#_Toc224126081)

[**3.11** NMR spectra of tris(pentafluoroethyl)phenylplumbane	44](#_Toc224126082)

[**3.12** NMR spectra of tris(pentafluoroethyl)tolylplumbane	47](#_Toc224126083)

[**3.13** NMR spectra of tris(pentafluoroethyl)anisylplumbane	50](#_Toc224126084)

[**3.14** NMR spectra of chlorotris(pentafluoroethyl)plumbane	52](#_Toc224126085)

[**3.15** NMR spectra of hexakis(pentafluoroethyl)diplumbane	54](#_Toc224126086)

[**3.16** NMR spectra of chlorotriphenylphosphonium-tris(pentafluoroethyl)plumbate	57](#_Toc224126087)

[**4 X-ray diffraction data**	60](#_Toc224126088)

[**5 References**	63](#_Toc224126089)

**1 General**

All chemicals were obtained from commercial sources and used without further purification. Standard high vacuum techniques were employed throughout all preparative procedures. Nonvolatile compounds were handled in a dry N_2_ atmosphere using Schlenk techniques. The NMR spectra were recorded on a Bruker Model Avance III 300 spectrometer (^1^H 300.13 MHz, ^19^F 282.40 MHz, ^31^P 121.49 MHz, ^13^C 75.47 MHz, ^207^Pb 62.79 MHz), a Bruker Model Avance III 500 (^1^H 500.01 MHz, ^19^F 470.48 MHz, ^31^P 202.41 MHz, ^13^C 128.73 MHz, ^207^Pb 104.61 MHz) or a Bruker Model Avance III 500 HD (^1^H 500.20 MHz, ^19^F 470.65 MHz, ^31^P 202.48 MHz, ^13^C 125.78 MHz, ^207^Pb 104.64 MHz) with positive shifts being downfield from the external standards (TMS (^13^C, ^1^H), CCl_3_F (^19^F), 85% H_3_PO_4_ (^31^P) and Pb(CH_3_)_4_ (^207^Pb)). IR spectra were recorded on an ALPHA-FT-IR spectrometer (Bruker Daltonik GmbH, Bremen, Germany) using a gas cell with KBr windows or an ATR unit with a diamond crystal for liquids and solids. The crystal data were collected using Mo Kα radiation (λ=71.073 pm). Using Olex2^[57]^, the structures were solved with the ShelXS^[58]^ structure solution program using direct methods and refined with the ShelXL^[58]^ refinement package using least-squares minimization. All atoms were refined anisotropically, except hydrogen atoms, which were refined isotropically. Details of the X-ray investigations are given in tables 1S and 2S. CCDC 2533633-2533639 contain the supplementary crystallographic data for this paper. These data can be obtained free of charge from the Cambridge Crystallographic Data Centre.

**2 Synthetic procedures**

**2.1** Synthesis of tetrakis(pentafluoroethyl)plumbane

*From Pb(C_2_F_5_)_3_Cl*: A *n*-butyllithium/*n*-pentane solution (1.3 mL, c = 2.0 mol · L^-1^, 2.6 mmol) was cooled to -78 °C, diluted with approx. 20 mL of dimethyl ether and stirred under an atmosphere of pentafluoroethane (3.5 mmol) for 20 min. Chlorotris(pentafluoroethyl)plumbane (1.3 g, 2.1 mmol) in *n*-pentane (10 mL) was added dropwise and the reaction mixture was allowed to warm to -25 °C. All volatile compounds were removed via fractional condensation and the product was collected in the −40 °C trap. Pb(C_2_F_5_)_4_ was obtained as a light yellow liquid (1.0 g, 1.5 mmol, 75 %).

*From Pb(OAc)_4_:* A *n*-butyllithium/*n*-pentane solution (24.0 mL, c = 2.0 mol · L^-1^, 48.0 mmol) was cooled to -78 °C, diluted with approx. 200 mL of dimethyl ether and stirred under an atmosphere of pentafluoroethane (57.2 mmol) for 20 min. Pb(OAc)_4_ (4.7 g, 10.6 mmol) was added and the reaction mixture was allowed to warm to room temperature (3 h). The resulting suspension was filtered and all volatile compounds were removed via fractional condensation, and the product was collected in the −40 °C trap. Pb(C_2_F_5_)_4_ was obtained as a light yellow liquid (2.0 g, 3.0 mmol, 28 %). ^13^C{^19^F} NMR (125 MHz, *n*-pentane/[D_6_]acetone, 25 °C): δ=143.6 (s, ^1^*J*(Pb,C)=480 Hz; CF_2_), 120.1 ppm (s, ^2^*J*(Pb,C)=162 Hz; CF_3_); ^19^F NMR (470 MHz, *n*-pentane/[D_6_]acetone, 25 °C): δ=-83.4 (s, ^1^*J*(F,C)=282 Hz, 12F; CF_3_), -95.0 ppm (s, ^1^*J*(F,C)=332 Hz, ^2^*J*(Pb,F)=651 Hz, 8F; CF_2_); ^207^Pb{^19^F} NMR (104 MHz, *n*-pentane/[D_6_]acetone, 25 °C): δ=-415.5 ppm (s; Pb); ^207^Pb NMR (104 MHz, *n*-pentane/[D_6_]acetone, 25 °C): δ=-415.5 ppm (non tridec, ^2^*J*(Pb,F)=653 Hz, ^3^*J*(Pb,F)=9 Hz; Pb); IR (gas phase): $\tilde{\nu}$=1313 (s, $\nu$[CC]), 1280 (w), 1224 (s, $\nu$[CF_3_]), 1131 (w, $\nu$[CF_2_]), 1102 (w, $\nu$[CF_2_]), 914 (m, $\nu$[PbC]), 740 (w, $\delta$[CF_3_]), 608 (vw), 536 (vw);MS (EI, 70 eV, pos.): m/z (%): 565 (10) [Pb(C_2_F_5_)_3_^+^], 446 (2) [Pb(C_2_F_5_)_2_^•+^], 327 (30) [Pb(C_2_F_5_)^+^], 227 (20) [PbF^+^], 208 (25) [Pb^•+^]; 100 (9) [C_2_F_4_^•+^], 69 (13) [CF_3_^+^], 43 (100).

**2.2** Synthesis of tetrakis(pentafluorethyl)-1,10-phenantrolinelead(IV)

Pb(C_2_F_5_)_4_ (1.0 g, 1.4 mmol) was condensed onto a solution of 1,10-phenanthroline (0.13 g, 0.7 mmol) dissolved *n*-pentane (8 mL). The reaction mixture was warmed to room temperature and an orange precipitate was formed. All volatile components were removed under reduced pressure and the phenanthroline complex [Pb(C_2_F_5_)_4_(phen)] remained as an orange solid (0.59 g, 0.68 mmol, 59 %). Crystals suitable for X-ray single crystal structure determination were grown by diffusing *n*-pentane in a cold chloroform solution. ^1^H NMR (300 MHz, CDCl_3_, -50 °C): δ=9.0 (s (br), 2H; H2/H9), 8.2 (d, ^3^*J*(H,H)=8 Hz, 2H; H4/H7), 7.7 (s, 2H; H5/H6), 7.5 ppm (d d, ^3^*J*(H,H)=8 Hz, ^3^*J*(H,H)=4 Hz; H3/H8); ^19^F NMR (282 MHz, CDCl_3_, -50 °C): δ=-80.8 (s, 6F; CF_3_), -81.9 (s, 6F; CF_3_), -101.0 (s, ^2^*J*(Pb,F)=995 Hz, 4F; CF_2_), -101.4 ppm (s, ^2^*J*(Pb,F)=404 Hz, 4F; CF_2_); ^207^Pb NMR (62 MHz, CDCl_3_, -50 °C): δ=-859.6 ppm (quin quin m, ^2^*J*(Pb,F)=1010 Hz, ^2^*J*(Pb,F)=404 Hz; Pb); ^19^F,^207^Pb-HMBC (282 MHz, CDCl_3_, -50 °C): δ=-80.8/-859.0 (s; CF_3_/Pb), -81.9 (s; CF_3_/Pb), -101.0 (d, ^2^*J*(Pb,F)=995 Hz; CF_2_/Pb), -101.4 ppm (d, ^2^*J*(Pb,F)=404 Hz; CF_2_/Pb).

**2.3** Synthesis of tetraphenylplumbane

LiPh was synthesized from lithium (3.5 g, 504.2 mmol) and bromobenzene (32.2 g 205.1 mmol) in diethyl ether (total 100 mL). Excess lithium and LiBr were removed and the solution was added dropwise to a solution of PbCl_2_ (10.0 g, 36.0 mmol) and iodobenzene (8.5 g, 51.7 mmol) in diethyl ether (50 mL). The reaction mixture was heated under reflux for 1 h. After cooling the mixture to 0 °C excess PhLi was hydrolysed with ice. The precipitate was filtered off and washed with water and diethyl ether. After drying in vacuo, PbPh_4_ was obtained as colourless solid (16.2 g, 31.4 mmol, 87 %). M.p. 227 – 230 °C; ^1^H NMR (500 MHz, [D_6_]DMSO, 25 °C): δ=7.6 (m, ^3^*J*(Pb,H)=80 Hz, 8H; H_ortho_), 7.5 (m, ^4^*J*(Pb,H)=26 Hz, 8H; H_meta_) , 7.4 ppm (m, ^5^*J*(Pb,H)=15 Hz, 4H; H_para_); ^13^C{^1^H} NMR (125 MHz, [D_6_]DMSO, 25 °C): δ=149.9 (s; C_ipso_), 137.6 (s, ^2^*J*(Pb,C)=67 Hz; C_ortho_), 130.2 (s, ^3^*J*(Pb,C)=79 Hz; C_meta_), 129.2 ppm (s; C_para_); ^207^Pb{^1^H} NMR (104 MHz, [D_6_]DMSO, 25 °C): δ=-187.6 ppm (s; Pb); MS (EI, 70 eV, pos.): m/z (%): 439 (100) [Pb(C_6_H_5_)_3_^+^], 285 (64) [Pb(C_6_H_5_)^+^], 208 (59) [Pb^•+^], 154 (13) [(C_6_H_5_)_2_^•+^], 78 (27) [C_6_H_5_^+^]; elemental analysis calcd (%) for C_24_H_20_Pb: C 55.91, H 3.91; found: C 56.00, H 3.84.

**2.4** Synthesis of tetratolylplumbane

LiTol was synthesized from lithium (3.1 g, 446.6 mmol) and 4-bromotoluene (33.7 g 197.0 mmol) in diethyl ether (total 170 mL). Excess lithium and LiBr were removed and the solution was added dropwise to a solution of PbCl_2_ (17.6 g, 63.3 mmol) and 4-iodotoluene (15.2 g, 69.7 mmol) in diethyl ether (60 mL). The reaction mixture was heated under reflux for 2 h. After cooling the mixture to 0 °C excess TolLi was hydrolysed with ice. The precipitate was filtered off and washed with ethanol. The precipitate was then solved in chloroform and filtered. After drying in vacuo, PbTol_4_ was obtained as colourless solid (32.9 g, 57.5 mmol, 91 %). M.p. 245 – 246 °C; ^1^H NMR (500 MHz, C_6_D_6_, 25 °C): δ=7.5 (d, ^3^*J*(H,H)=8 Hz, ^3^*J*(Pb,H)=80 Hz, 8H; H_ortho_), 7.2 (d, ^3^*J*(H,H)=8 Hz, ^4^*J*(Pb,H)=24 Hz, 8H; H_meta_), 2.4 ppm (s, ^6^*J*(Pb,H)=5 Hz, 12H; CH_3_); ^13^C{^1^H} NMR (125 MHz, C_6_D_6_, 25 °C): δ=146.4 (s, ^1^*J*(Pb,C)=493 Hz; C_ipso_), 138.2 (s, ^4^*J*(Pb,C)=18 Hz; C_para_), 137.5 (s, ^2^*J*(Pb,C)=70 Hz; C_ortho_), 130.2 (s, ^3^*J*(Pb,C)=83 Hz; C_meta_), 21.5 ppm (s, ^5^*J*(Pb,C)=5 Hz; CH_3_); ^207^Pb{^1^H} NMR (62 MHz, C_6_D_6_, 25 °C): δ=-167.9 ppm (s; Pb); MS (EI, 70 eV, pos.): m/z (%): 481 (100) [Pb(C_7_H_7_)_3_^+^], 299 (45) [Pb(C_7_H_7_)^+^], 208 (40) [Pb^•+^], 182 (15) [(C_7_H_7_)_2_^•+^], 91 (15) [C_7_H_7_^+^]; elemental analysis calcd (%) for C_28_H_28_Pb: C 58.82, H 4.94; found: C 58.74, H 4.99.

**2.5** Synthesis of tetraanisylplumbane

LiAni was synthesized from lithium (2.8 g, 403.4 mmol) and 4-bromoanisole (24.1 g, 128.9 mmol) in diethyl ether (115 mL). Excess lithium and LiBr were removed and the solution was added dropwise to a solution of PbCl_2_ (9.2 g, 33.1 mmol) and 4-iodoanisole (8.5 g, 36.2 mmol) in diethyl ether (40 mL). The reaction mixture was heated under reflux for 2 h. After cooling the mixture to 0 °C excess AniLi was hydrolysed with ice. The precipitate was filtered off and the solvent of the filtrate was removed under reduced pressure. The combined solids were solved in dichloromethane dried, over magnesiumsulfate and filtered. After drying in vacuo, PbAni_4_ was obtained as beige solid (20.6 g, 32.4 mmol, 98 %). M.p. 140°C; ^1^H NMR (500 MHz, C_6_D_6_, 25 °C): δ=7.7 (d m, ^3^*J*(H,H)=8 Hz, ^3^*J*(Pb,H)=77 Hz, 8H; H_ortho_), 6.9 (d, ^3^*J*(H,H)=8 Hz, ^4^*J*(Pb,H)=19 Hz, 8H; H_meta_), 3.3 ppm (s, 12H; OCH_3_); ^13^C{^1^H} NMR (125 MHz, C_6_D_6_, 25 °C): δ=160.3 (s, ^4^*J*(Pb,C)=19 Hz; C_para_), 140.5 (s, ^1^*J*(Pb,C)=520 Hz; C_ipso_), 138.6 (s, ^2^*J*(Pb,C)=79 Hz; C_ortho_), 115.5 (s, ^3^*J*(Pb,C)=88 Hz; C_meta_), 54.3 ppm (s; OCH_3_); ^207^Pb{^1^H} NMR (62 MHz, C_6_D_6_, 25 °C): δ=-145.1 ppm (s, ^1^*J*(Pb,C)=520 Hz, ^3^*J*(Pb,C)=88 Hz, ^2^*J*(Pb,C)=79 Hz, ^4^*J*(Pb,C)=19 Hz; Pb); MS (EI, 70 eV, pos.): m/z (%): 635 (1) [Pb(C_7_H_7_O)_4_^•+^], 529 (100) [Pb(C_7_H_7_O)_3_^+^], 315 (51) [Pb(C_7_H_7_O)^+^], 214 (70) [(C_7_H_7_O)_2_^•+^], 208 (30) [Pb^•+^], 199 (35) [(C_7_H_7_O)(C_6_H_4_O)^+^], 108 (22) [C_7_H_8_O^+^]; elemental analysis calcd (%) for C_28_H_28_O_4_Pb: C 52.90, H 4.44; found: C 50.63, H 4.34.

**2.6** Synthesis of bis(pentafluoroethyl)diphenylplumbane

Hydrogen chloride (51.5 mmol) was condensed onto a cooled (-78 °C) solution of PbPh_4_ (10.1 g, 19.6 mmol) in dichloromethane (60 mL) and slowly warmed to room temperature. All volatile compounds were removed in vacuo. Dichlorodiphenylplumbane was obtained as a colourless solid (8.6 g, 19.8 mmol, 101 %).

A *n*-butyllithium/*n*-hexane solution (36.0 mL, c = 1.6 mol · L^-1^, 57.6 mmol) was diluted with 200 mL of diethyl ether, cooled to -78 °C, degassed and stirred under an atmosphere of pentafluoroethane (66.3 mmol) for 20 min. Dichlorodiphenylplumbane (8.2 g, 19.0 mmol) was added and the reaction mixture was allowed to warm to ambient temperature (3 h). The resulting suspension was filtered and all volatile compounds were removed from the filtrate under reduced pressure. The obtained crude product was purified by vacuum distillation (3∙10^-2^ mbar, b.p.: 122 °C). Pb(C_2_F_5_)_2_Ph_2_ was obtained as a colourless liquid (9.7 g, 16.1 mmol, 85 %). ^1^H NMR (300 MHz, neat/[D_6_]acetone, 25 °C): δ=6.9 (d, ^3^*J*(H,H)=8 Hz, ^3^*J*(Pb,H)=110 Hz, 4H; H_ortho_), 6.7 (d d, ^3^*J*(H,H)=8 Hz, ^3^*J*(H,H)=8 Hz, ^4^*J*(Pb,H)=41 Hz, 4H; H_meta_), 6.6 ppm (d, ^3^*J*(H,H)=8 Hz, ^5^*J*(Pb,H)=21 Hz, 2H; H_para_); ^13^C{^1^H} NMR (75 MHz, C6D6, 25 °C): δ=147.5 (quin, ^3^*J*(F,C)=3 Hz, ^1^*J*(Pb,C)=592 Hz; C_ipso_), 139.1 (t q, ^1^*J*(F,C)=319 Hz, ^2^*J*(F,C)=50 Hz, ^1^*J*(Pb,C)=183 Hz; CF_2_), 136.8 (s, ^2^*J*(Pb,C)=86 Hz; C_ortho_), 130.8 (s, ^3^*J*(Pb,C)=109 Hz; C_meta_), 130.6 (s, ^4^*J*(Pb,C)=25 Hz; C_para_), 121.5 ppm (q t, ^1^*J*(F,C)=283 Hz, ^2^*J*(F,C)=27 Hz, ^2^*J*(Pb,C)=100 Hz; CF_3_); ^13^C{^19^F} NMR (75 MHz, C6D6, 25 °C): δ=147.5 (t, ^2^*J*(C,H)=9 Hz, ^1^*J*(Pb,C)=592 Hz; C_ipso_), 139.1 (s, ^1^*J*(Pb,C)=183 Hz; CF_2_), 136.8 (d m, ^1^*J*(C,H)=164 Hz, ^2^*J*(Pb,C)=86 Hz; C_ortho_), 130.8 (d t m, ^1^*J*(C,H)=162 Hz, ^2^*J*(C,H)=10 Hz, ^3^*J*(Pb,C)=109 Hz; C_meta_), 130.6 (d t m, ^1^*J*(C,H)=160 Hz, ^2^*J*(C,H)=8 Hz, ^4^*J*(Pb,C)=25 Hz; C_para_), 121.5 ppm (s, ^2^*J*(Pb,C)=100 Hz; CF_3_); ^19^F NMR (282 MHz, neat/[D_6_]acetone, 25 °C): δ=-83.9 (s, ^1^*J*(F,C)=283 Hz, ^2^*J*(F,C)=27 Hz, 6F; CF_3_), -106.8 ppm (s, ^2^*J*(Pb,F)=408 Hz, 4F; CF_2_); ^207^Pb{^1^H} NMR (62 MHz, neat/[D_6_]acetone, 25 °C): δ=-249.1 ppm (quin, ^2^*J*(Pb,F)=408 Hz; Pb); ^207^Pb{^19^F} NMR (62 MHz, neat/[D_6_]acetone, 25 °C): δ=-249.1 ppm (quin quin t, ^3^*J*(Pb,H)=110 Hz, ^4^*J*(Pb,H)=42 Hz, ^5^*J*(Pb,H)=21 Hz; Pb); MS (EI, 70 eV, pos.): m/z (%): 481 (59) [Pb(C_2_F_5_)(C_6_H_5_)_2_^+^], 285 (41) [Pb(C_6_H_5_)^+^], 208 (100) [Pb^•+^], 154 (13) [(C_6_H_5_)_2_^•+^], 119 (5) [C_2_F_5_^+^], 78 (27) [C_6_H_5_^+^], 69 (5) [CF_3_^+^]; elemental analysis calcd (%) for C_16_H_10_F_10_Pb: C 32.06, H 1.68; found: C 32.16, H 1.30.

**2.7** Synthesis of bis(pentafluoroethyl)ditolylplumbane

Hydrogen chloride (130 mmol) was condensed onto a cooled (-78 °C) solution of PbTol_4_ (29.3 g, 51.2 mmol) in dichloromethane (250 mL) and slowly warmed to room temperature. All volatile compoundes were removed in vacuo. Dichloroditolylplumbane was obtained as a colourless solid (23.6 g, 51.3 mmol, 100 %).

A *n*-butyllithium/*n*-hexane solution (90.0 mL, c = 1.6 mol · L^-1^, 144.0 mmol) was diluted with 300 mL of diethyl ether, cooled to -78 °C, degassed and stirred under an atmosphere of pentafluoroethane (190 mmol) for 20 min. Dichloroditolylplumbane (23.0 g, 50.0 mmol) was added and the reaction mixture was allowed to warm to ambient temperature (over night). The resulting suspension was filtered and all volatile compounds were removed from the filtrate under reduced pressure. The obtained crude product was purified by vacuum distillation (1∙10^-3^ mbar, b.p.: 108 °C). Pb(C_2_F_5_)_2_Tol_2_ was obtained as a colourless liquid (29.7 g, 47.3 mmol, 95 %). ^1^H NMR (300 MHz, neat/[D_6_]acetone, 25 °C): δ=6.9 (d, ^3^*J*(H,H)=8 Hz, ^3^*J*(Pb,H)=110 Hz, 4H; H_ortho_), 6.6 (d, ^3^*J*(H,H)=8 Hz, ^4^*J*(Pb,H)=40 Hz, 4H; H_meta_), 1.6 ppm (s, ^1^*J*(C,H)=128 Hz, ^6^*J*(Pb,H)=7 Hz, 6H; CH_3_); ^13^C{^1^H} NMR (75 MHz, neat/[D_6_]acetone, 25 °C): δ=143.2 (m, ^1^*J*(Pb,C)=609 Hz; C_ipso_), 140.3 (s, ^4^*J*(Pb,C)=25 Hz; C_para_), 137.9 (t q, ^1^*J*(F,C)=320 Hz, ^2^*J*(F,C)=50 Hz; CF_2_), 135.6 (s, ^2^*J*(Pb,C)=90 Hz; C_ortho_), 130.6 (s, ^3^*J*(Pb,C)=115 Hz; C_meta_), 120.6 (q t, ^1^*J*(F,C)=282 Hz, ^2^*J*(F,C)=27 Hz; CF_3_), 19.6 ppm (s, ^5^*J*(Pb,C)=14 Hz; CH_3_); ^13^C{^19^F} NMR (75 MHz, neat/[D_6_]acetone, 25 °C): δ=147.3 (t, ^2^*J*(C,H)=17 Hz, ^1^*J*(Pb,C)=609 Hz; C_ipso_), 140.3 (t q, ^2^*J*(C,H)=13 Hz, ^2^*J*(C,H)=6 Hz; C_para_), 137.9 (s, ^1^*J*(Pb,C)=180 Hz; CF_2_), 135.6 (d m, ^1^*J*(C,H)=159 Hz, ^2^*J*(Pb,C)=90 Hz; C_ortho_), 130.6 (d m, ^1^*J*(C,H)=158 Hz; C_meta_), 120.6 (s, ^2^*J*(Pb,C)=97 Hz; CF_3_), 19.6 ppm (q t, ^1^*J*(C,H)=126 Hz, ^3^*J*(C,H)=4 Hz; CH_3_); ^19^F NMR (282 MHz, neat/[D_6_]acetone, 25 °C): δ=-83.8 (s, ^1^*J*(F,C)=283 Hz, ^2^*J*(F,C)=27 Hz, 6F; CF_3_), -107.2 ppm (s, ^2^*J*(Pb,F)=398 Hz, ^1^*J*(F,C)=319 Hz, 4F; CF_2_); ^207^Pb{^1^H} NMR (62 MHz, neat/[D_6_]acetone, 25 °C): δ=-233.1 ppm (quin, ^2^*J*(Pb,F)=400 Hz; Pb); ^207^Pb{^19^F} NMR (62 MHz, neat/[D_6_]acetone, 25 °C): δ=-233.1 ppm (quin quin sept, ^3^*J*(Pb,H)=110 Hz, ^4^*J*(Pb,H)=40 Hz, ^6^*J*(Pb,H)=7 Hz; Pb); MS (EI, 70 eV, pos.): m/z (%): 509 (60) [Pb(C_2_F_5_)(C_7_H_7_)_2_^+^], 299 (32) [Pb(C_7_H_7_)^+^], 208 (100) [Pb^•+^], 182 (18) [(C_7_H_7_)_2_^•+^], 91 (87) [C_7_H_7_^+^], 69 (21) [CF_3_^+^]; elemental analysis calcd (%) for C_18_H_14_F_10_Pb: C 34.45, H 2.25; found: C 34.42, H 2.45.

**2.8** Synthesis of bis(pentafluoroethyl)dianisylplumbane

Hydrogen chloride (56.0 mmol) was condensed onto a cooled (-78 °C) solution of PbAni_4_ (11.3 g, 17.8 mmol) in dichloromethane (50 mL) and slowly warmed to room temperature. All volatile compounds were removed in vacuo. Dichlorodianisylplumbane was obtained as a reddish solid (9.0 g, 18.1 mmol, 102 %).

A *n*-butyllithium/*n*-hexane solution (30.0 mL, c = 1.6 mol · L^-1^, 48.0 mmol) was diluted with 200 mL of diethyl ether, cooled to -78 °C, degassed and stirred under an atmosphere of pentafluoroethane (59 mmol) for 20 min. Dichlorodianisylplumbane (8.2 g, 16.7 mmol) was added and the reaction mixture was allowed to warm to ambient temperature (3 h). The resulting suspension was filtered and all volatile compounds were removed from the filtrate under reduced pressure. The obtained crude product was purified by vacuum distillation (1∙10^-3^ mbar, b.p.: 145 °C). Pb(C_2_F_5_)_2_Ani_2_ was obtained as a colourless liquid (8.4 g, 12.7 mmol, 76 %). ^1^H NMR (300 MHz, neat/[D_6_]acetone, 25 °C): δ=7.0 (d, ^3^*J*(H,H)=8 Hz, ^3^*J*(Pb,H)=105 Hz, 4H; H_ortho_), 6.4 (d, ^3^*J*(H,H)=8 Hz, ^4^*J*(Pb,H)=32 Hz, 4H; H_meta_), 3.0 ppm (s, ^1^*J*(C,H)=144 Hz, 6H; OCH_3_); ^13^C{^1^H} NMR (75 MHz, neat/[D_6_]acetone, 25 °C): δ=161.0 (s, ^4^*J*(Pb,C)=26 Hz; C_para_), 137.6 (t q, ^1^*J*(F,C)=316 Hz, ^2^*J*(F,C)=47 Hz; CF_2_), 137.0 (s, ^1^*J*(Pb,C)=647 Hz; C_ipso_), 136.9 (s, ^2^*J*(Pb,C)=101 Hz; C_ortho_), 120.6 (q t, ^1^*J*(F,C)=282 Hz, ^2^*J*(F,C)=54 Hz; CF_3_), 115.6 (s, ^3^*J*(Pb,C)=124 Hz; C_meta_), 54.3 ppm (s; OCH_3_); ^13^C{^19^F} NMR (75 MHz, neat/[D_6_]acetone, 25 °C): δ=161.0 (m; C_para_), 137.6 (s, ^1^*J*(Pb,C)=191 Hz; CF_2_), 137.0 (t, ^2^*J*(C,H)=9 Hz; C_ipso_), 136.9 (d d, ^1^*J*(C,H)=162 Hz, ^2^*J*(C,H)=9 Hz, ^2^*J*(Pb,C)=101 Hz; C_ortho_), 120.6 (s, ^2^*J*(Pb,C)=99 Hz; CF_3_), 115.6 (d d, ^1^*J*(C,H)=161 Hz, ^2^*J*(C,H)=4 Hz, ^3^*J*(Pb,C)=124 Hz; C_meta_), 53.4 ppm (q, ^1^*J*(C,H)=144 Hz; OCH_3_); ^19^F NMR (282 MHz, neat/[D_6_]acetone, 25 °C): δ=-83.8 (s, ^1^*J*(F,C)=283 Hz, 6F; CF_3_), -107.7 ppm (s, ^2^*J*(Pb,F)=400 Hz, 4F; CF_2_); ^207^Pb{^1^H} NMR (62 MHz, neat/[D_6_]acetone, 25 °C): δ=-217.9 ppm (quin, ^2^*J*(Pb,F)=400 Hz; Pb); ^207^Pb{^19^F} NMR (62 MHz, neat/[D_6_]acetone, 25 °C): δ=-217.9 ppm (quin quin, ^3^*J*(Pb,H)=105 Hz, ^4^*J*(Pb,H)=33 Hz; Pb); MS (EI, 70 eV, pos.): m/z (%): 659 (<1) [Pb(C_2_F_5_)_2_(C_7_H_7_O)_2_^•+^], 541 (45) [Pb(C_2_F_5_)(C_7_H_7_O)_2_^+^], 315 (25) [Pb(C_7_H_7_O)^+^], 214 (100) [(C_7_H_7_O)_2_^•+^], 208 (45) [Pb^•+^], 199 (25) [C_13_H_11_O^+^]; elemental analysis calcd (%) for C_18_H_14_F_10_O_2_Pb: C 32.78, H 2.14; found: C 32.00, H 2.08.

**2.9** Synthesis of (pentafluoroethyl)triphenylplumbane

Hydrogen chloride (20.0 mmol) was condensed onto a cooled (-78 °C) solution of PbPh_4_ (10.5 g, 20.4 mmol) in dichloromethane (200 mL) and slowly warmed to room temperature. All volatile compoundes were removed in vacuo. Chlorotriphenylplumbane was obtained as a colourless solid (9.5 g, 20.0 mmol, 98 %).

A *n*-butyllithium/*n*-hexane solution (17.0 mL, c = 1.6 mol · L^-1^, 27.2 mmol) was diluted with 250 mL of diethyl ether, cooled to -78 °C, degassed and stirred under an atmosphere of pentafluoroethane (33.75 mmol) for 20 min. Chlorotriphenylplumbane (9.0 g, 19.0 mmol) was added and the reaction mixture was allowed to warm to ambient temperature (3 h). All volatile compounds were removed under reduced pressure. The residue was extracted with dichloromethane (150 mL) and filtrated. The solvent was removed under reduced pressure. The obtained crude product was washed with cold *n*-pentane (3 x 10 mL). After drying in vacuo Pb(C_2_F_5_)Ph_3_ was obtained as a white crystalline solid (6.5 g, 10.1 mmol, 50 %). From the combined *n*-pentane solutions Pb(C_2_F_5_)_2_Ph_2_ was obtained as a colourless liquid (2.9 g, 4.8 mmol, 25 %) by removing the solvent. Pb(C_2_F_5_)Ph_3_: ^1^H NMR (500 MHz, CD_2_Cl_2_, 25 °C): δ=7.7 (d, ^3^*J*(H,H)=7 Hz, ^3^*J*(Pb,H)=93 Hz, 6H; H_ortho_), 7.6 (d d, ^3^*J*(H,H)=7 Hz, ^3^*J*(H,H)=7 Hz, ^4^*J*(Pb,H)=32 Hz, 6H; H_meta_), 7.5 ppm (d, ^3^*J*(H,H)=7 Hz, ^5^*J*(Pb,H)=17 Hz, 3H; H_para_); ^13^C{^1^H} NMR (125 MHz, CD_2_Cl_2_, 25 °C): δ=148.7 (t, ^3^*J*(F,C)=3 Hz, ^1^*J*(Pb,C)=545 Hz; C_ipso_), 137.3 (s, ^2^*J*(Pb,C)=75 Hz; C_ortho_), 135.2 (t q, ^1^*J*(F,C)=318 Hz, ^2^*J*(F,C)=50 Hz; CF_2_), 130.1 (s, ^3^*J*(Pb,C)=94 Hz; C_meta_), 129.7 (s, ^4^*J*(Pb,C)=24 Hz; C_para_), 121.6 ppm (q t, ^1^*J*(F,C)=283 Hz, ^2^*J*(F,C)=27 Hz; CF_3_); ^13^C{^19^F} NMR (125 MHz, CD_2_Cl_2_, 25 °C): δ=148.7 (t m, ^2^*J*(C,H)=8 Hz, ^1^*J*(Pb,C)=545 Hz; C_ipso_), 137.3 (d m, ^1^*J*(C,H)=164 Hz, ^2^*J*(Pb,C)=75 Hz; C_ortho_), 135.2 (s, ^1^*J*(Pb,C)=52 Hz; CF_2_), 130.8 (d m, ^1^*J*(C,H)=162 Hz, ^3^*J*(Pb,C)=94 Hz; C_meta_), 129.7 (d m, ^1^*J*(C,H)=161 Hz, ^4^*J*(Pb,C)=24 Hz; C_para_), 121.6 ppm (s, ^2^*J*(Pb,C)=74 Hz; CF_3_); ^19^F NMR (470 MHz, CD_2_Cl_2_, 25 °C): δ=-82.6 (s, ^1^*J*(F,C)=283 Hz, ^2^*J*(F,C)=48 Hz, ^3^*J*(Pb,F)=6 Hz, 3F; CF_3_), -110.6 ppm (s, ^1^*J*(F,C)=318 Hz, ^2^*J*(F,C)=28 Hz, ^2^*J*(Pb,F)=324 Hz, 2F; CF_2_); ^207^Pb{^1^H} NMR (104 MHz, CD_2_Cl_2_, 25 °C): δ=-204.1 ppm (t q, ^2^*J*(Pb,F)=320 Hz, ^3^*J*(Pb,F)=6 Hz; Pb); ^207^Pb{^19^F} NMR (104 MHz, CD_2_Cl_2_, 25 °C): δ=-204.1 ppm (sept sept t, ^3^*J*(Pb,H)=93 Hz, ^4^*J*(Pb,H)=32 Hz, ^5^*J*(Pb,H)=17 Hz; Pb); MS (EI, 70 eV, pos.): m/z (%): 439 (100) [Pb(C_6_H_5_)_3_^+^], 285 (70) [Pb(C_6_H_5_)^+^], 208 (65) [Pb^•+^], 154 (7) [(C_6_H_5_)_2_^•+^], 77(27) [C_6_H_5_^+^]; elemental analysis calcd (%) for C_20_H_15_F_5_Pb: C 43.09, H 2.71; found: C 43.60, H 2.76.

**2.10** Synthesis of (pentafluoroethyl)tritolylplumbane

Hydrogen chloride (9.5 mmol) was condensed onto a cooled (-78 °C) solution of PbTol_4_ (5.5g, 9.6 mmol) in dichloromethane (100 mL) and slowly warmed to room temperature. All volatile compounds were removed in vacuo. Chlorotritolylplumbane was obtained as a colourless solid (4.6 g, 8.9 mmol, 94 %).

A *n*-butyllithium/*n*-hexane solution (10.0 mL, c = 1.6 mol · L^-1^, 16.0 mmol) was diluted with 100 mL of diethyl ether, cooled to -78 °C, degassed and stirred under an atmosphere of pentafluoroethane (20.0 mmol) for 20 min. Chlorotritolylplumbane (4.3 g, 8.3 mmol) was added and the reaction mixture was allowed to warm to ambient temperature (3 h). The resulting suspension was filtered and all volatile compounds were removed from the filtrate under reduced pressure. The obtained crude product was washed with *n*-pentane (3 x 30 mL). After drying in vacuo Pb(C_2_F_5_)Tol_3_ was obtained as a white crystalline solid (1.2 g, 2.0 mmol, 25 %). From the combined *n*-pentane solutions Pb(C_2_F_5_)_2_Tol_2_ was obtained as a colourless liquid (1.0 g, 1.6 mmol, 20 %) by removing the solvent. Pb(C_2_F_5_)Tol_3_: M.p. 135 °C; ^1^H NMR (500 MHz, CDCl_3_, 25 °C): δ=7.7 (d, ^3^*J*(H,H)=8 Hz, ^3^*J*(Pb,H)=93 Hz, 6H; H_ortho_), 7.2 (d, ^3^*J*(H,H)=8 Hz, ^4^*J*(Pb,H)=31 Hz, 6H; H_meta_), 2.3 ppm (s, ^1^*J*(C,H)=127 Hz, ^6^*J*(Pb,H)=5 Hz, 9H; CH_3_); ^13^C{^1^H} NMR (125 MHz, CDCl_3_, 25 °C): δ=143.9 (t, ^3^*J*(F,C)=6 Hz, ^1^*J*(Pb,C)=556 Hz; C_ipso_), 138.5 (s, ^4^*J*(Pb,C)=22 Hz; C_para_), 136.0 (s, ^2^*J*(Pb,C)=80 Hz; C_ortho_), 135.1 (t q, ^1^*J*(F,C)=317 Hz, ^2^*J*(F,C)=48 Hz; CF_2_), 129.7 (s, ^3^*J*(Pb,C)=96 Hz; C_meta_), 120.4 (q t, ^1^*J*(F,C)=283 Hz, ^2^*J*(F,C)=27 Hz; CF_3_), 20.3 ppm (s, ^5^*J*(Pb,C)=12 Hz; CH_3_); ^19^F NMR (470 MHz, CDCl_3_, 25 °C): δ=-82.3 (s, ^1^*J*(F,C)=283 Hz, ^2^*J*(F,C)=48 Hz, ^3^*J*(Pb,F)=6 Hz, 3F; CF_3_), -110.4 ppm (s, ^1^*J*(F,C)=317 Hz, ^2^*J*(Pb,F)=329 Hz, 2F; CF_2_); ^207^Pb{^1^H} NMR (104 MHz, CDCl_3_, 25 °C): δ=-187.1 ppm (t q, ^2^*J*(Pb,F)=329 Hz, ^3^*J*(Pb,F)=6 Hz; Pb); ^207^Pb{^19^F} NMR (62 MHz, C_6_D_6_, 25 °C): δ=-181.0 ppm (sept sept dec, ^3^*J*(Pb,H)=93 Hz, ^4^*J*(Pb,H)=31 Hz, ^6^*J*(Pb,H)=6 Hz; Pb); MS (EI, 70 eV, pos.): m/z (%): 481 (100) [Pb(C_7_H_7_)_3_^+^], 299 (56) [Pb(C_7_H_7_)^+^], 208 (65) [Pb^•+^], 91 (10) [C_7_H_7_^+^]; elemental analysis calcd (%) for C_23_H_21_F_5_Pb: C 46.07, H 3.53; found: C 46.52, H 3.71.

**2.11** Synthesis of tris(pentafluoroethyl)phenylplumbane

Hydrogen chloride (32.3 mmol) was condensed onto a cooled (-78 °C) solution of Pb(C_2_F_5_)_2_Ph_2_ (8.6 g, 14.4 mmol) in dichloromethane (50 mL) and slowly warmed to room temperature. All volatile compounds were removed in vacuo. Chlorobis(pentafluoroethyl)phenylplumbane was obtained as a colourless solid (7.5 g, 13.4 mmol, 93 %).

A *n*-butyllithium/*n*-hexane solution (16.0 mL, c = 1.6 mol · L^-1^, 25.6 mmol) was diluted with 100 mL of diethyl ether, cooled to -78 °C, degassed and stirred under an atmosphere of pentafluoroethane (30.0 mmol) for 20 min. Chlorobis(pentafluoroethyl)phenylplumbane (6.8 g, 12.1 mmol) was added and the reaction mixture was allowed to warm to ambient temperature (over night). The resulting suspension was filtered and all volatile compounds were removed from the filtrate under reduced pressure. The obtained crude product was purified by vacuum distillation (20∙mbar, b.p.: 91 °C). Pb(C_2_F_5_)_3_Ph was obtained as a colourless liquid (7.0 g, 10.9 mmol, 90 %). ^1^H NMR (300 MHz, CDCl_3_, 25 °C): δ=7.6 (t, ^3^*J*(H,H)=8 Hz, ^4^*J*(Pb,H)=54 Hz, 2H; H_meta_), 7.5 (t t, ^3^*J*(H,H)=8 Hz, ^5^*J*(Pb,H)=25 Hz, 1H; H_para_), 7.4 ppm (d, ^3^*J*(H,H)=8 Hz, ^3^*J*(Pb,H)=131 Hz, 2H; H_ortho_); ^13^C{^1^H} NMR (75 MHz, neat/[D_6_]acetone, 25 °C): δ=146.5 (quin, ^3^*J*(F,C)=4 Hz, ^1^*J*(Pb,C)=620 Hz; C_ipso_), 140.9 (t q m, ^1^*J*(F,C)=322 Hz, ^2^*J*(F,C)=51 Hz; CF_2_), 135.1 (s, ^2^*J*(Pb,C)=94 Hz; C_ortho_), 130.6 (s, ^4^*J*(Pb,C)=30 Hz; C_para_), 130.3 (s, ^3^*J*(Pb,C)=129 Hz; C_meta_), 120.0 ppm (q t, ^1^*J*(F,C)=282 Hz, ^2^*J*(F,C)=27 Hz; CF_3_); ^13^C{^19^F} NMR (75 MHz, neat/[D_6_]acetone, 25 °C): δ=146.5 (t m, ^2^*J*(C,H)=9 Hz, ^1^*J*(Pb,C)=620 Hz; C_ipso_), 140.9 (s, ^1^*J*(Pb,C)=341 Hz; CF_2_), 135.1 (d m, ^1^*J*(C,H)=165 Hz, ^2^*J*(Pb,C)=94 Hz; C_ortho_), 130.6 (d t, ^1^*J*(C,H)=162 Hz, ^2^*J*(C,H)=8 Hz; C_para_), 130.3 (d m, ^1^*J*(C,H)=163 Hz, ^3^*J*(Pb,C)=129 Hz; C_meta_), 120.0 ppm (s, ^2^*J*(Pb,C)=130 Hz; CF_3_); ^19^F NMR (282 MHz, neat/[D_6_]acetone, 25 °C): δ=-84.5 (s, ^1^*J*(F,C)=282 Hz, 9F; CF_3_), ^-1^02.1 ppm (s, ^2^*J*(Pb,F)=515 Hz, ^1^*J*(F,C)=323 Hz, 6F; CF_2_); ^207^Pb{^1^H} NMR (62 MHz, neat/[D_6_]acetone, 25 °C): δ=-318.5 ppm (sep dec, ^2^*J*(Pb,F)=515 Hz, ^3^*J*(Pb,F)=5 Hz; Pb); ^207^Pb{^19^F} NMR (62 MHz, neat/[D_6_]acetone, 25 °C): δ=-318.5 ppm (t t d, ^3^*J*(Pb,H)=133 Hz, ^4^*J*(Pb,H)=53 Hz, ^5^*J*(Pb,H)=25 Hz; Pb); ^207^Pb NMR (62 MHz, neat/[D_6_]acetone, 25 °C): δ=-318.5 ppm (sep t t d dec, ^2^*J*(Pb,F)=515 Hz, ^3^*J*(Pb,H)=133 Hz, ^4^*J*(Pb,H)=53 Hz, ^5^*J*(Pb,H)=25 Hz, ^3^*J*(Pb,F)=5 Hz; Pb); MS (EI, 20 eV, pos.): m/z (%): 523 (75) [Pb(C_2_F_5_)_2_(C_6_H_5_)^+^], 285 (100) [Pb(C_6_H_5_)^+^], 208 (27) [Pb^•+^], 119 (40) [C_2_F_5_^+^], 69 (5) [CF_3_^+^]; elemental analysis calcd (%) for C_12_H_5_F_15_Pb: C 22.47, H 0.79; found: C 22.25, H 0.80.

**2.12** Synthesis of tris(pentafluoroethyl)tolylplumbane

Hydrogen chloride (100 mmol) was condensed onto a cooled (-78 °C) solution of Pb(C_2_F_5_)_2_Tol_2_ (29.0 g, 46.2 mmol) in dichloromethane (200 mL) and slowly warmed to room temperature. All volatile compounds were removed in vacuo. Chlorobis(pentafluoroethyl)tolylplumbane was obtained as a light yellowish solid (25.7 g, 45.0 mmol, 97 %).

A *n*-butyllithium/*n*-hexane solution (40.0 mL, c = 1.6 mol · L^-1^, 64.0 mmol) was diluted with 250 mL of diethyl ether, cooled to -78 °C, degassed and stirred under an atmosphere of pentafluoroethane (77 mmol) for 20 min. Chlorobis(pentafluorethyl)tolylplumbane (25.2 g, 44.1 mmol) was added and the reaction mixture was allowed to warm to ambient temperature (over night). The resulting suspension was filtered and all volatile compounds were removed from the filtrate under reduced pressure. The obtained crude product was purified by vacuum distillation (7∙mbar, b.p.: 92 °C). Pb(C_2_F_5_)_3_Tol was obtained as a colourless liquid (27.0 g, 41.2 mmol, 94 %). ^1^H NMR (300 MHz, neat/[D_6_]acetone, 25 °C): δ=7.04 (d, ^3^*J*(H,H)=8 Hz, ^3^*J*(Pb,H)=130 Hz, 2H; H_ortho_), 6.95 (d, ^3^*J*(H,H)=8 Hz, ^4^*J*(Pb,H)=50 Hz, 3H; H_meta_), 1.9 ppm (s, ^1^*J*(C,H)=128 Hz, ^6^*J*(Pb,H)=9 Hz, 3H; CH_3_); ^13^C{^1^H} NMR (75 MHz, neat/[D_6_]acetone, 25 °C): δ=143.0 (sep, ^3^*J*(F,C)=4 Hz, ^1^*J*(Pb,C)=639 Hz; C_ipso_), 141.8 (s, ^4^*J*(Pb,C)=31 Hz; C_para_), 137.9 (t q m, ^1^*J*(F,C)=322 Hz, ^2^*J*(F,C)=51 Hz; CF_2_), 134.9 (s, ^2^*J*(Pb,C)=99 Hz; C_ortho_), 131.1 (s, ^3^*J*(Pb,C)=135 Hz; C_meta_), 120.1 (q t, ^1^*J*(F,C)=282 Hz, ^2^*J*(F,C)=27 Hz; CF_3_), 19.3 ppm (s, ^5^*J*(Pb,C)=18 Hz; CH_3_); ^13^C{^19^F} NMR (75 MHz, neat/[D_6_]acetone, 25 °C): δ=143.0 (t, ^2^*J*(C,H)=9 Hz, ^1^*J*(Pb,C)=639 Hz; C_ipso_), 141.8 (q, ^2^*J*(C,H)=6 Hz; C_para_), 141.0 (s, ^1^*J*(Pb,C)=336 Hz; CF_2_), 134.9 (d d, ^1^*J*(C,H)=164 Hz, ^2^*J*(C,H)=8 Hz, ^2^*J*(Pb,C)=90 Hz; C_ortho_), 131.1 (d m, ^1^*J*(C,H)=160 Hz, ^3^*J*(Pb,C)=135 Hz; C_meta_), 120.1 (s, ^2^*J*(Pb,C)=130 Hz; CF_3_), 19.6 ppm (q t, ^1^*J*(C,H)=127 Hz, ^3^*J*(C,H)=4 Hz; CH_3_); ^19^F NMR (282 MHz, neat/[D_6_]acetone, 25 °C): δ=-84.5 (s, ^1^*J*(F,C)=282 Hz, 9F; CF_3_), -102.3 ppm (s, ^2^*J*(Pb,F)=511 Hz, ^1^*J*(F,C)=323 Hz, 6F; CF_2_); ^207^Pb{^1^H} NMR (62 MHz, neat/[D_6_]acetone, 25 °C): δ=-306.3 ppm (sep dec, ^2^*J*(Pb,F)=510 Hz, ^3^*J*(Pb,F)=4 Hz; Pb); ^207^Pb{^19^F} NMR (62 MHz, neat/[D_6_]acetone, 25 °C): δ=-306.3 ppm (t t q, ^3^*J*(Pb,H)=132 Hz, ^4^*J*(Pb,H)=51 Hz, ^6^*J*(Pb,H)=9 Hz; Pb); ^207^Pb NMR (62 MHz, neat/[D_6_]acetone, 25 °C): δ=-306.3 ppm (sep t t q dec, ^2^*J*(Pb,F)=510 Hz, ^3^*J*(Pb,H)=132 Hz, ^4^*J*(Pb,H)=51 Hz, ^6^*J*(Pb,H)=9 Hz, ^3^*J*(Pb,F)=4 Hz; Pb); MS (EI, 70 eV, pos.): m/z (%): 655 (< 1) [Pb(C_2_F_5_)_3_(C_7_H_7_)^•+^], 537 (56) [Pb(C_2_F_5_)_2_(C_7_H_7_)^+^], 299 (100) [Pb(C_7_H_7_)^+^], 208 (21) [Pb^•+^]; elemental analysis calcd (%) for C_13_H_7_F_15_Pb: C 23.82, H 1.08; found: C 23.82, H 1.14.

## **2.13** Synthesis of tris(pentafluoroethyl)anisylplumbane

Hydrogen chloride (7.5 mmol) was condensed onto a cooled (-78 °C) solution of Pb(C_2_F_5_)_2_Ani_2_ (2.2 g, 3.3 mmol) in dichloromethane (20 mL) and slowly warmed to room temperature. All volatile compounds were removed in vacuo. Chlorobis(pentafluoroethyl)anisylplumbane was obtained as a beige solid (1.7 g, 3.0 mmol, 90 %).

An *n*-butyllithium/*n*-hexane solution (3.7 mL, c = 1.6 mol · L^-1^, 5.9 mmol) was diluted with 30 mL of diethyl ether, cooled to -78 °C, degassed and stirred under an atmosphere of pentafluoroethane (8.8 mmol) for 20 min. Chlorobis(pentafluorethyl)anisylplumbane (1.4 g, 2.3 mmol) was added and the reaction mixture was allowed to warm to ambient temperature (3 h). The resulting suspension was filtered and all volatile compounds were removed from the filtrate under reduced pressure. The obtained crude product was purified by vacuum condensation (1∙10^-3^ mbar). Pb(C_2_F_5_)_3_Ani was obtained as a colourless liquid (1.5 g, 2.2 mmol, 95 %). ^1^H NMR (500 MHz, C_6_D_6_ , 25 °C): δ=7.1 (d, ^3^*J*(H,H)=8 Hz, ^3^*J*(Pb,H)=125 Hz, 2H; H_ortho_), 6.6 (d, ^3^*J*(H,H)=8 Hz, ^4^*J*(Pb,H)=44 Hz, 2H; H_meta_), 3.1 ppm (s, 3H; OCH_3_); ^13^C{^1^H} NMR (125 MHz, C_6_D_6_, 25 °C): δ= 162.0 (s, ^4^*J*(Pb,C)=31 Hz; C_para_), 141.3 (t q m, ^1^*J*(F,C)=323 Hz, ^2^*J*(F,C)=51 Hz; CF_2_),136.8 (m, ^1^*J*(Pb,C)=670 Hz; C_ipso_), 136.7 (s, ^2^*J*(Pb,C)=115 Hz; C_ortho_), 121.7 (q t, ^1^*J*(F,C)=282 Hz, ^2^*J*(F,C)=28 Hz; CF_3_), 117.0 (s, ^3^*J*(Pb,C)=145 Hz; C_meta_), 54.3 ppm (s; OCH_3_); ^13^C{^19^F} NMR (125 MHz, C_6_D_6_, 25 °C): δ=162.0 (m; C_ipso_), 141.3 (s, ^1^*J*(Pb,C)=334 Hz; CF_2_), 136.8 (t, ^2^*J*(C,H)=9 Hz, ^1^*J*(Pb,C)=670 Hz; C_ipso_), 134.9 (d d, ^1^*J*(C,H)=164 Hz, ^2^*J*(C,H)=8 Hz, ^2^*J*(Pb,C)=90 Hz; C_ortho_), 131.1 (d m, ^1^*J*(C,H)=160 Hz, ^3^*J*(Pb,C)=135 Hz; C_meta_), 120.1 (s, ^2^*J*(Pb,C)=130 Hz; CF_3_), 19.6 ppm (q t, ^1^*J*(C,H)=127 Hz, ^3^*J*(C,H)=4 Hz; OCH_3_); ^19^F NMR (470 MHz, C_6_D_6_, 25 °C): δ=-82.9 (s, ^1^*J*(F,C)=282 Hz, 9F; CF_3_), -101.2 ppm (s, ^2^*J*(Pb,F)=508 Hz, ^1^*J*(F,C)=323 Hz, 6F; CF_2_); ^207^Pb{^1^H} NMR (104 MHz, C_6_D_6_, 25 °C): δ=-292.1 ppm (sep dec, ^2^*J*(Pb,F)=508 Hz, ^3^*J*(Pb,F)=4 Hz; Pb); ^207^Pb{^19^F} NMR (104 MHz, C_6_D_6_, 25 °C): δ=-292.1 ppm (t t, ^3^*J*(Pb,H)=125 Hz, ^4^*J*(Pb,H)=45 Hz; Pb); MS (EI, 70 eV, pos.): m/z (%): 671 (< 1) [Pb(C_2_F_5_)_3_(C_7_H_7_O)^•+^] 553 (36) [Pb(C_2_F_5_)_2_(C_7_H_7_O)^+^], 315 (100) [Pb(C_7_H_7_O)^+^], 208 (40) [Pb^•+^]; elemental analysis calcd (%) for C_13_H_7_F_15_OPb: C 23.26, H 1.05; found: C 23.61, H 1.15.

## **2.14** Synthesis of chlorotris(pentafluoroethyl)plumbane

Hydrogen chloride (21.3 mmol) was condensed onto neat Pb(C_2_F_5_)_3_Tol (5.0 g, 7.6 mmol) and warmed to room temperature. The reaction mixture was stirred for 2 days. Yellow crystals sublimed on the wall of the reaction vessel. The product was purified by cold trap condensation (1∙10^-3^ mbar, -10 °C). Pb(C_2_F_5_)_3_Cl was obtained as a yellow crystalline solid (4.7 g, 7.8 mmol, 102 %). Vapour pressure (25 °C)=6∙mbar; ^13^C{^19^F} NMR (75 MHz, *n*-pentane/C_6_D_6_, 25 °C): δ=143.3 (s, ^1^*J*(Pb,C)=674 Hz; CF_2_), 119.6 ppm (s, ^2^*J*(Pb,C)=211 Hz; CF_3_); ^19^F NMR (282 MHz, *n*-pentane/C_6_D_6_, 25 °C): δ=-82.5 (s, ^1^*J*(F,C)=282 Hz, ^3^*J*(Pb,F)=23 Hz, 9F; CF_3_), -94.6 ppm (s, ^1^*J*(F,C)=336 Hz, ^2^*J*(Pb,F)=817 Hz, 6F; CF_2_); ^207^Pb NMR (62 MHz, *n*-pentane/C_6_D_6_, 25 °C): δ=-246f.3 ppm (sep, ^2^*J*(Pb,F)=817 Hz; Pb); IR (gas phase): $\tilde{\nu}$=1330 (w, $\nu_{s}$[CC]), 1313 (s, $\nu_{as}$[CC]), 1256 (w), 1229 (s, $\nu$[CF_3_]), 1149 (m, $\nu_{as}$[CF_2_]), 1105 (w, $\nu_{s}$[CF_2_]), 904 (m, $\nu$[PbC]), 739 (w, $\delta$[CF_3_]), 609 (w, $\delta$[CF_2_]), 535 (w, $\delta_{as}$[CF_3_]); MS (EI, 20 eV, pos.): m/z (%): 565 (2) [Pb(C_2_F_5_)_3_^+^], 446 (1) [Pb(C_2_F_5_)_2_^•+^], 362 (1) [Pb(C_2_F_5_)Cl^•+^], 327 (5) [Pb(C_2_F_5_)^+^], 243 (4) [PbCl^+^], 208 (21) [Pb^•+^], 119 (43) [C_2_F_5_^+^], 101 (31) [C_2_F_4_H^+^], 100 (11) [C_2_F_4_^•+^], 69 (100) [CF_3_^+^], 51 (51) [Pb(CF_2_H)^+^].

## **2.15** Synthesis of hexakis(pentafluorethyl)diplumbane

Pb(C_2_F_5_)_3_Cl (0.51 g, 0.85 mmol) was condensed onto a suspension of SbF_3_ (0.18 g, 1.0 mmol) in *n-*pentane (10 mL) and slowly warmed to room temperature. The reaction mixture was stirred for 2 h under exclusion of light. The precipitate was filtered off and volatile compounds were removed *in vacuo*. After high vacuum sublimation Pb_2_(C_2_F_5_)_6_ was obtained as a yellow solid that partly melts at room temperature. (0.26 g, 0.25 mmol, 54 %). ^19^F NMR (282 MHz, *n*-pentane/C_6_D_6_, 25 °C): δ=-82.6 (s, ^1^*J*(F,C)=287 Hz, 18F; CF_3_), -93.9 ppm (s, ^1^*J*(F,C)=335 Hz, ^2^*J*(Pb,F)=600 Hz, ^3^*J*(Pb,F)=59 Hz, 8F; CF_2_); ^13^C{^19^F} NMR (75 MHz, *n*-pentane/C_6_D_6_, 25 °C): δ=145.0 (s, ^1^*J*(Pb,C)=174 Hz, ^2^*J*(Pb,C)=108 Hz; CF_2_), 120.3 ppm (s, ^2^*J*(Pb,C)=139 Hz, ^3^*J*(Pb,C)=24 Hz; CF_3_); ^207^Pb{^19^F} NMR (62 MHz, *n*-pentane/C_6_D_6_, 25 °C): δ=16.6 ppm (s; Pb); ^207^Pb{^19^F(CF_3_)} NMR (62 MHz, *n*-pentane/C_6_D_6_, 25 °C): δ=16.6 ppm (sept sept, ^2^*J*(Pb,F)=593 Hz, ^3^*J*(Pb,F)=59 Hz; Pb); ^207^Pb NMR (62 MHz, *n*-pentane/C_6_D_6_, 25 °C): δ=16.6 ppm (sept sept dec, ^2^*J*(Pb,F)=593 Hz, ^3^*J*(Pb,F)=59 Hz, ^3^*J*(Pb,F)=15 Hz; Pb); MS (EI, 20 eV, pos.): m/z (%): 1012 (<1) [Pb_2_(C_2_F_5_)_5_^+^], 892 (<1) [Pb_2_(C_2_F_5_)_4_^+^], 789 (1) [Pb_2_(C_2_F_5_)_3_F^+^], 773 (<1) [Pb_2_(C_2_F_5_)_3_^+^], 655 (<1) [Pb_2_(C_2_F_5_)_2_^+^], 565 (1) [Pb(C_2_F_5_)_3_^+^], 327 (15) [PbC_2_F_5_^+^], 226 (10) [PbF^+^], 208 (15) [Pb^•+^], 119 (43) [C_2_F_5_^+^], 100 (55) [C_2_F_4_^•+^], 69 (100) [CF_3_^+^].

## **2.16** Synthesis of chloro­triphenyl­phosphonium­-tris(pentafluoroethyl)­plumbate

Pb(C_2_F_5_)_3_Cl (1.4 g, 2.4 mmol) was condensed onto a suspension of PPh_3_ (0.6 g, 2.4 mmol) in *n-*pentane (10 mL) and slowly warmed to room temperature. The reaction mixture was stirred for 10 min. The solvent was decanted and the white precipitate was washed with *n-*pentane (10 mL). [PPh_3_Cl][Pb(C_2_F_5_)_3_] was obtained as a white fluffy solid (1.9 g, 2.2 mmol, 92 %). ^1^H NMR (300 MHz, CD_2_Cl_2_, 25 °C): δ=8.1 – 7.3 (m, H_arom_); ^13^C{^1^H} NMR (75 MHz, CD_2_Cl_2_, 25 °C): δ=137.4 (d, ^4^*J*(P,C)=3 Hz; C_para_), 133.6 (d, ^3^*J*(P,C)=13 Hz; C_meta_), 130.9 (d, ^2^*J*(fP,C)= 15 Hz; C_ortho_), 118.7 ppm (d, ^1^*J*(P,C)=93 Hz; C_ipso_); ^13^C{^19^F} NMR (75 MHz, CD_2_Cl_2_, 25 °C): δ=172.3 (s, ^1^*J*(Pb,C)=1390 Hz; CF_2_), 124.7 ppm (s, ^2^*J*(Pb,C)=27 Hz; CF_3_); ^19^F NMR (282 MHz, CD_2_Cl_2_, 25 °C): δ=-82.9 (t quin, ^3^*J*(F,F)=2 Hz, ^5^*J*(F,F)=2 Hz, ^1^*J*(F,C)=282 Hz ^3^*J*(Pb,F)=61 Hz, 9F; CF_3_), -113.8 ppm (q sep, ^3^*J*(F,F)=2 Hz, ^5^*J*(F,F)=2 Hz, ^1^*J*(F,C)=326 Hz, ^2^*J*(Pb,F)=42 Hz, 6F; CF_2_); ^31^P{^1^H} NMR (121 MHz, CD_2_Cl_2_, 25 °C): δ= 66 ppm (s, ^1^*J*(P,C)=93 Hz, ^2^*J*(P,C)= 15 Hz, ^3^*J*(P,C)=13 Hz, ^4^*J*(P,C)=3 Hz; P); ^207^Pb{^19^F} NMR (62 MHz, CD_2_Cl_2_, 25 °C): δ=1608.8 ppm (s, ^1^*J*(Pb,C)=1390 Hz; Pb); ^207^Pb NMR (62 MHz, CD_2_Cl_2_, 25 °C): δ=1608.8 ppm (dec sep, ^3^*J*(Pb,F)=61 Hz, ^2^*J*(Pb,F)=42 Hz, ^1^*J*(Pb,C)=1390 Hz; Pb).

**3 NMR spectra**

**3.1** NMR spectra of tetrakis(pentafluoroethyl)plumbane

**Figure S1.** ^13^C{^19^F} NMR spectrum of Pb(C_2_F_5_)_4_ in *n*-pentane/[D_6_]acetone.

**Figure S2.** ^19^F NMR spectrum Pb(C_2_F_5_)_4_ in *n*-pentane/[D_6_]acetone.

**Figure S3.** ^207^Pb{^1^H} NMR spectrum of Pb(C_2_F_5_)_4_ in *n*-pentane/[D_6_]acetone.

**Figure S4.** ^207^Pb{^19^F} NMR spectrum of Pb(C_2_F_5_)_4_ in *n*-pentane/[D_6_]acetone.

## **3.2** NMR spectra of tetrakis(pentafluoroethyl)-1,10-phenantrolinelead(IV)

**Figure S5.** ^1^H NMR spectrum of P(C_2_F_5_)_4_(phen) in CDCl_3_ at −50 °C.

**Figure S6.** ^19^F NMR spectrum of of P(C_2_F_5_)_4_(phen) in CDCl_3_ at −50 °C.

**Figure S7.** ^19^F^207^Pb HMBC NMR spectrum of P(C_2_F_5_)_4_(phen) in CDCl_3_ at −50 °C.

**Figure S8.** ^207^Pb NMR spectrum of P(C_2_F_5_)_4_(phen) in CDCl_3_ at −50 °C.

**3.3** NMR spectra of tetraphenylplumbane

**Figure S9.** ^1^H NMR spectrum of PbPh_4_ in [D_6_]DMSO. *H_2_O.

**Figure S10.** ^13^C{^1^H} NMR spectrum of PbPh_4_ in [D_6_]DMSO.

**Figure S11.** ^207^Pb NMR spectrum of PbPh_4_ in [D_6_]DMSO.

**3.4** NMR spectra of tetratolylplumbane

**Figure S12.** ^1^H NMR Spectrum of PhTol_4_ in C_6_D_6_.

**Figure S13.** ^207^Pb{^1^H} NMR Spectrum of PhTol_4_ in C_6_D_6_.

## **3.5** NMR spectra of tetraanisylplumbane

**Figure S14.** ^1^H NMR Spectrum of PbAni_4_ in C_6_D_6_.

**Figure S15.** ^13^C{^1^H} NMR Spectrum of PbAni_4_ in C_6_D_6_.

**Figure S16.** ^207^Pb NMR Spectrum of PbAni_4_ in C_6_D_6_.

## **3.6** NMR spectra of bis(pentafluoroethyl)diphenylplumbane

**Figure S17.** ^1^H NMR spectrum of neat Pb(C_2_F_5_)_2_Ph_2_ with [D_6_]acetone as external reference.

**Figure S18.** ^19^F NMR spectrum of neat Pb(C_2_F_5_)_2_Ph_2_ with [D_6_]acetone as external reference.

**Figure S19.** ^207^Pb{^1^H} NMR spectrum of neat Pb(C_2_F_5_)_2_Ph_2_ with [D_6_]acetone as external reference.

**Figure S20.** ^207^Pb{^19^F} NMR spectrum of neat Pb(C_2_F_5_)_2_Ph_2_ with [D_6_]acetone as external reference.

## **3.7** NMR spectra of bis(pentafluoroethyl)ditolylplumbane

**Figure S21.** ^1^H NMR spectrum of neat Pb(C_2_F_5_)_2_Tol_2_ with [D_6_]acetone as external reference.

**Figure S22.** ^13^C{^1^H} NMR spectrum of neat Pb(C_2_F_5_)_2_Tol_2_ with [D_6_]acetone (*) as external reference.

**Figure S23.** ^13^C{^19^F} NMR spectrum of neat Pb(C_2_F_5_)_2_Tol_2_ with [D_6_]acetone (*) as external reference.

**Figure S24.** ^19^F NMR spectrum of neat Pb(C_2_F_5_)_2_Tol_2_ with [D_6_]acetone as external reference.

**Figure S25.** ^207^Pb{^1^H} NMR spectrum of neat Pb(C_2_F_5_)_2_Tol_2_ with [D_6_]acetone as external reference.

**Figure S26.** ^207^Pb{^19^F} NMR spectrum of neat Pb(C_2_F_5_)_2_Tol_2_ with [D_6_]acetone as external reference.

## **3.8** NMR spectra of bis(pentafluoroethyl)dianisylplumbane

**Figure S27.** ^1^H NMR spectrum of neat Pb(C_2_F_5_)_2_Ani_2_ with [D_6_]acetone as external reference.

**Figure S28.** ^13^C{^1^H} NMR spectrum of neat Pb(C_2_F_5_)_2_Ani_2_ with [D_6_]acetone (*) as external reference.

**Figure S29.** ^13^C{^19^F} NMR spectrum of neat Pb(C_2_F_5_)_2_Ani_2_ with [D_6_]acetone (*) as external reference.

**Figure S30.** ^19^F NMR spectrum of neat Pb(C_2_F_5_)_2_Ani_2_ with [D_6_]acetone as external reference.

**Figure S31.** ^207^Pb{^1^H} NMR spectrum of neat Pb(C_2_F_5_)_2_Ani_2_ with [D_6_]acetone as external reference.

**Figure S32.** ^207^Pb{^19^F} NMR spectrum of neat Pb(C_2_F_5_)_2_Ani_2_ with [D_6_]acetone as external reference.

## **3.9** NMR spectra of (pentafluoroethyl)triphenylplumbane

**Figure S33.** ^1^H NMR spectrum of Pb(C_2_F_5_)Ph_3_ in CD_2_Cl_2_. *H_2_O.

**Figure S34.** ^13^C{^19^F} NMR spectrum of Pb(C_2_F_5_)Ph_3_ in CD_2_Cl_2_.

**Figure S35.** ^19^F NMR spectrum of Pb(C_2_F_5_)Ph_3_ in CD_2_Cl_2_.

**Figure S36.** ^207^Pb{^1^H} NMR spectrum of Pb(C_2_F_5_)Ph_3_ in CD_2_Cl_2_.

**Figure S37.** ^207^Pb{^19^F} NMR spectrum of Pb(C_2_F_5_)Ph_3_ in CD_2_Cl_2_.

## **3.10** NMR spectra of (pentafluoroethyl)tritolylplumbane

**Figure S38.** ^1^H NMR spectrum of Pb(C_2_F_5_)Tol_3_ in CDCl_3_.

**Figure S39.** ^13^C{^19^F} NMR spectrum of Pb(C_2_F_5_)Tol_3_ in CDCl_3_.

**Figure S40.** ^19^F NMR spectrum of Pb(C_2_F_5_)Tol_3_ in CDCl_3_.

**Figure S41.** ^207^Pb{^19^F} NMR spectrum of Pb(C_2_F_5_)Tol_3_ in CDCl_3_.

## **3.11** NMR spectra of tris(pentafluoroethyl)phenylplumbane

**Figure S42.** ^1^H NMR spectrum of Pb(C_2_F_5_)_3_Ph in CDCl_3_.

**Figure S43.** ^13^C{^1^H} NMR spectrum of neat Pb(C_2_F_5_)_3_Ph with [D_6_]acetone (*) as external reference.

**Figure S44.** ^13^C{^19^F} NMR spectrum of neat Pb(C_2_F_5_)_3_Ph with [D_6_]acetone (*) as external reference.

**Figure S45.** ^19^F NMR spectrum of neat Pb(C_2_F_5_)_3_Ph with [D_6_]acetone as external reference.

**Figure S46.** ^207^Pb NMR spectrum of neat Pb(C_2_F_5_)_3_Ph with [D_6_]acetone as external reference.

**Figure S47.** ^207^Pb{^1^H} NMR spectrum of neat Pb(C_2_F_5_)_3_Ph with [D_6_]acetone as external reference.

## **3.12** NMR spectra of tris(pentafluoroethyl)tolylplumbane

**Figure S48.** 1H NMR spectrum of neat Pb(C_2_F_5_)_3_Tol with [D_6_]acetone as external reference.

**Figure S49.** ^13^C{^1^H} NMR spectrum of neat Pb(C_2_F_5_)_3_Tol with [D_6_]acetone (*) as external reference.

**Figure S50.** ^13^C{^19^F} NMR spectrum of neat Pb(C_2_F_5_)_3_Tol with [D_6_]acetone (*) as external reference.

**Figure S51.** ^19^F NMR spectrum of neat Pb(C_2_F_5_)_3_Tol with [D_6_]acetone as external reference.

**Figure S52.** ^207^Pb{^1^H} NMR spectrum of neat Pb(C_2_F_5_)_3_Tol with [D_6_]acetone as external reference.

**Figure S53.** ^207^Pb{^19^F} NMR spectrum of neat Pb(C_2_F_5_)_3_Tol with [D_6_]acetone as external reference.

## **3.13** NMR spectra of tris(pentafluoroethyl)anisylplumbane

**Figure S54.** ^1^H NMR spectrum of Pb(C_2_F_5_)_3_Ani in C_6_D_6_.

**Figure S55.** ^13^C{^19^F} NMR spectrum of Pb(C_2_F_5_)_3_Ani in C_6_D_6_.

**Figure S56.** ^19^F NMR spectrum of Pb(C_2_F_5_)_3_Ani in C_6_D_6_.

**Figure S57.** ^207^Pb{^1^H} NMR spectrum of Pb(C_2_F_5_)_3_Ani in C_6_D_6_.

**Figure S58.** ^207^Pb{^19^F} NMR spectrum of Pb(C_2_F_5_)_3_Ani in C_6_D_6_.

## **3.14** NMR spectra of chlorotris(pentafluoroethyl)plumbane

**Figure S59.** ^13^C{^19^F} NMR spectrum of Pb(C_2_F_5_)_3_Cl in *n*-pentane/C_6_D_6_.

**Figure S60.** ^19^F NMR spectrum of Pb(C_2_F_5_)_3_Cl in *n*-pentane/C_6_D_6_.

**Figure S61.** ^207^Pb NMR spectrum of Pb(C_2_F_5_)_3_Cl in *n*-pentane/C_6_D_6_.

## **3.15** NMR spectra of hexakis(pentafluoroethyl)diplumbane

**Figure S62.** ^13^C{^19^F} NMR spectrum of Pb_2_(C_2_F_5_)_6_ in *n*-pentane/C_6_D_6_.

**Figure S63.** ^19^F NMR spectrum of Pb_2_(C_2_F_5_)_6_ in *n*-pentane/C_6_D_6_.

**Figure S64.** ^207^Pb NMR spectrum of Pb_2_(C_2_F_5_)_6_ in *n*-pentane/C_6_D_6_.

**Figure S65.** ^207^Pb{^19^F} NMR spectrum of Pb_2_(C_2_F_5_)_6_ in *n*-pentane/C_6_D_6_.

**Figure S66.** ^207^Pb{^19^F[CF_3_]} NMR spectrum of Pb_2_(C_2_F_5_)_6_ in *n*-pentane/C_6_D_6_.

## **3.16** NMR spectra of chlorotriphenylphosphonium-tris(pentafluoroethyl)plumbate

**Figure S67.** ^1^H NMR spectrum of [PPh_3_Cl][Pb(C_2_F_5_)_3_] in CD_2_Cl_2_.

**Figure S68.** ^13^C{^1^H} NMR spectrum of [PPh_3_Cl][Pb(C_2_F_5_)_3_] in CD_2_Cl_2_.

**Figure S69.** ^13^C{^19^F} NMR spectrum of [PPh_3_Cl][Pb(C_2_F_5_)_3_] in CD_2_Cl_2_.

**Figure S70.** ^19^F NMR spectrum of [PPh_3_Cl][Pb(C_2_F_5_)_3_] in CD_2_Cl_2_.

**Figure S71.** ^31^P{^1^H} NMR spectrum of [PPh_3_Cl][Pb(C_2_F_5_)_3_] in CD_2_Cl_2_.

**Figure S72.** ^207^Pb NMR spectrum of [PPh_3_Cl][Pb(C_2_F_5_)_3_] in CD_2_Cl_2_.

**Figure S73.** ^207^Pb{^19^F} NMR spectrum of [PPh_3_Cl][Pb(C_2_F_5_)_3_] in CD_2_Cl_2_.

**4 X-ray diffraction data**

**Table S1**: X-ray diffraction data for Pb(C_2_F_5_)_3_Cl, [Pb(C_2_F_5_)_4_(phen)] and Pb_2_(C_2_F_5_)_6_.

|  | Pb(C_2_F_5_)_3_Cl | [Pb(C_2_F_5_)_4_(phen)] | Pb_2_(C_2_F_5_)_6_ |
| --- | --- | --- | --- |
| empirical formula | C_6_ClF_15_Pb | C_20_H_8_F_20_N_2_Pb | C_6_F_15_Pb |
| *a* / pm | 1484.56(3) | 963.760(18) | 997.429(20) |
| *b* / pm | 1484.56(3) | 1354.29(3) | 1368.093(16) |
| *c* / pm | 1056.59(2) | 1855.15(3) | 956.406(14) |
| *α* / ° | 90 | 90 | 90 |
| *β* / ° | 90 | 104.9106(418) | 113.879(2) |
| *γ* / ° | 90 | 90 | 90 |
| *V* / 10^6^ ∙ pm^3^ | 2016.66(8) | 2339.82(7) | 1193.37(4) |
| Z | 6 | 4 | 4 |
| ρ_calc_ / mg∙mm^-3^ | 2.963 | 2.451 | 3.141 |
| crystal system | trigonal | monoclinic | monoclinic |
| space group | *R*3*c* | *P*2_1_/*c* | *P*2_1_/*c* |
| shape / colour | yellow fragment | yellow fragment | yellow fragment |
| crystal size / mm^3.^ | 0.17 x 0.11 x 0.06 | 0.25 x 0.23 x 0.11 | 0.35 x 0.25 x 0.16 |
| μ / mm^‑1^ | 12.918 | 7.386 | 14.326 |
| *T* / K | 99.99(11) | 100.01(10) | 100.0(1) |
| F(000) | 1620.0 | 1616.0 | 1012.0 |
| 2θ range for data  collection / ° | 5.488 to 60.046 | 5.314 to 60.16 | 4.466 to 60.068 |
| index ranges | −20 ≤ h ≤ 20 −20 ≤ k ≤ 20 −14 ≤ l ≤ 14 | −13 ≤ h ≤ 13 −15 ≤ k ≤ 19 −26 ≤ l ≤ 26 | −14 ≤ h ≤ 14 −19 ≤ k ≤ 19 −13 ≤ l ≤ 13 |
| reflections collected | 22802 | 39404 | 68024 |
| independent reflections [R(int)] | 1319  [0.0424] | 6875  [0.0376] | 3481  [0.0487] |
| data/restraints/  pa­ra­me­ters | 1319/1/71 | 6875/0/388 | 3481/0/199 |
| goodness-of-fit on F^2^ | 1.034 | 1.039 | 1.164 |
| R_1_ / wR_2_ [I≤2σ(I)] | 0.0100 / 0.0216 | 0.0199 / 0.0436 | 0.0160 / 0.0344 |
| R_1_ / wR_2_ (all data) | 0.0160 / 0.0233 | 0.0252 / 0.0459 | 0.0177 / 0.0350 |
| Δρ_max/min_ / e Å^-3^ | 0.72 / −0.38 | 0.17 / −1.63 | 1.49 / -0.70 |
| Flack parameter | 0.513(12) |  |  |
| CCDC number | 2533633 | 2533634 | 2533635 |

**Table S2:** X-ray diffraction data for [PPh_3_Cl][Pb(C_2_F_5_)_3_], Pb(C_2_F_5_)Tol_3_, Pb(C_2_F_5_)Ph_3_ and PbAni_4._

|  | [PPh_3_Cl] [Pb(C_2_F_5_)_3_] | Pb(C_2_F_5_)Tol_3_ | Pb(C_2_F_5_)Ph_3_ | PbAni_4_ |
| --- | --- | --- | --- | --- |
| empirical formula | C_24_H_15_ClF_15_PPb | C_23_H_21_F_5_Pb | C_20_H_15_F_5_Pb | C_28_H_28_O_4_Pb |
| *a* / pm | 1156.166(18) | 1192.455(14) | 1017.104(18) | 1421.78(1) |
| *b* / pm | 1416.956(9) | 978.127(11) | 1280.62(2) | 1421.78(1) |
| *c* / pm | 1695.070(11) | 1851.96(2) | 1487.25(2) | 629.548(11) |
| *α* / ° | 90 | 90 | 90.6190(15) | 90 |
| *β* / ° | 90 | 90.6250(10) | 108.2998(16) | 90 |
| *γ* / ° | 90 | 90 | 93.7843(15) | 90 |
| *V* / 10^6^ ∙ pm^3^ | 2776.93(3) | 2159.95(4) | 1834.17(6) | 1272.49(4) |
| Z | 4 | 4 | 4 | 2 |
| ρ_calc_ / mg∙mm^-3^ | 2.062 | 1.844 | 2.019 | 1.659 |
| crystal system | orthorhombic | monoclinic | triclinic | tetragonal |
| space group | *P*2_1_2_1_2_1_ | *P*2_1_/*n* | *P*-1 | *I*-4 |
| shape / colour | colourless fragment | colourless fragment | colourless fragment | colourless fragment |
| crystal size / mm^3.^ | 0.42 x 0.22 x 0.17 | 0.29 x 0.25 x 0.1 | 0.25 x 0.18 x 0.15 | 0.27 x 0.14 x 0.07 |
| μ / mm^‑1^ | 6.345 | 7.859 | 9.246 | 6.659 |
| *T* / K | 100.00(11) | 100.00(11) | 100.0(1) | 99.98(13) |
| F(000) | 1632.0 | 1144.0 | 1048.0 | 620.0 |
| 2θ range for data  collection / ° | 3.746 to 60.148 | 4.042 to 60.0 | 5.58 to 65.864 | 4.052 to 59.988 |
| index ranges | −16 ≤ h ≤ 16 −19 ≤ k ≤ 19 −23 ≤ l ≤ 23 | −16 ≤ h ≤ 16 −13 ≤ k ≤ 13 −26 ≤ l ≤ 26 | −15 ≤ h ≤ 15 −19 ≤ k ≤ 19 −21 ≤ l ≤ 22 | −20 ≤ h ≤ 20 −20 ≤ k ≤ 20 −8 ≤ l ≤ 8 |
| reflections collected | 162740 | 114368 | 58306 | 37542 |
| independent reflections [R(int)] | 8145  [0.0482] | 6310  [0.0525] | 12676  [0.0401] | 1854  [0.0307] |
| data/restraints/  pa­ra­me­ters | 8145/0/379 | 6310/0/265 | 12676/0/597 | 1854/0/76 |
| goodness-of-fit on F^2^ | 1.080 | 1.044 | 1.046 | 1.153 |
| R_1_ / wR_2_ [I≤2σ(I)] | 0.0152 /  0.0387 | 0.0175 /  0.0393 | 0.0225 /  0.0466 | 0.0090 /  0.0229 |
| R_1_ / wR_2_ (all data) | 0.0154 / 0.0388 | 0.0221 / 0.0411 | 0.0337 / 0.0489 | 0.0090 / 0.0229 |
| Δρ_max/min_ / e Å^-3^ | 1.35 / −0.70 | 1.18 / −1.09 | 1.60 / −1.89 | 0.60 / −0.16 |
| Flack parameter | -0.0095(11) |  |  | −0.023(2) |
| CCDC number | 2533636 | 2533637 | 2533638 | 2533639 |

**5 References**

[57] O. V. Dolomanov, L. J. Bourhis, R. J. Gildea, J. A. K. Howard, H. Puschmann, *J. Appl. Cryst.* **2009**, *42(*2), 339–341.

[58]	G. M. Sheldrick, *Acta Cryst. A* **2008**, *64(*1), 112–122.
